# Growth of Gram-Negative Bacteria in Antiseptics, Disinfectants and Hand Hygiene Products in Two Tertiary Care Hospitals in West Africa—A Cross-Sectional Survey

**DOI:** 10.3390/pathogens12070917

**Published:** 2023-07-07

**Authors:** Palpouguini Lompo, Anne-Sophie Heroes, Esenam Agbobli, Adama Kazienga, Marjan Peeters, Halidou Tinto, Katrien Lagrou, Lassana Sangaré, Dissou Affolabi, Jan Jacobs

**Affiliations:** 1 Clinical Research Unit of Nanoro, Institut de Recherche en Science de la Santé, Nanoro, Ouagadougou 11 BP 218, Burkina Faso; 2 Department of Clinical Sciences, Institute of Tropical Medicine, Nationalestraat 155, 2000 Antwerp, Belgiumjjacobs@itg.be (J.J.); 3 Department of Microbiology, Immunology and Transplantation, KU Leuven, Naamsestraat 22 Box 5401, 3000 Leuven, Belgium; 4 Centre National Hospitalier Universitaire Hubert Koutoukou Maga, Cotonou 01 BP 386, Benin; 5 Centre Hospitalier Universitaire Yalgado Ouédraogo, Ouagadougou 03 BP 7022, Burkina Faso

**Keywords:** antiseptics, bacteria, cross-sectional, disinfectants, Gram-negative, hand hygiene, low-and middle-income countries, healthcare-associated infections, resistance, West Africa

## Abstract

Antiseptics, disinfectants, and hand hygiene products can act as reservoirs of Gram-negative bacteria causing healthcare-associated infections. This problem is rarely documented in low- and middle-income countries, particularly in sub-Saharan Africa. In a cross-sectional survey, we assessed the bacterial contamination of antiseptics, disinfectants, and hand hygiene products in two university hospitals in Burkina Faso and Benin. During ward visits and staff interviews, in-use products were cultured for the presence of Gram-negative bacteria. The growth of Gram-negative bacteria was absent or rare in alcohol-based products, povidone iodine, and Dakin solution. Contamination was highest (73.9% (51/69)) for liquid soap products (versus antiseptic/disinfectants (4.5%, 7/157) (*p* < 0.0001)), mostly used in high-risk areas and associated with high total bacterial counts (>10,000 colony-forming units/mL). Contaminating flora (105 isolates) included Enterobacterales and the *Vibrio* non-cholerae/*Aeromonas* group (17.1%) and non-fermentative Gram-negative rods (82.8%). Multidrug resistance was present among 9/16 Enterobacterales (*Klebsiella* and *Enterobacter* spp.) and 3/12 *Acinetobacter* spp., including carbapenem resistance (*Acinetobacter baumannii*: NDM, *Pseudomonas stutzeri*: VIM). The risk factors for contamination included the type of product (cleaning grade and in-house prepared liquid soap), use of recycled disposable containers and soft drink bottles, absence of labeling, topping-up of containers, dilution with tap water (pharmacy and ward), and poor-quality management (procurement, stock management, expiry dates, and period after opening).

## 1. Introduction

Antimicrobial resistance (AMR) is a worldwide health problem, and low- and middle-income countries (LMIC) are the hardest hit [1,2]. Among the factors contributing to AMR in LMIC, poor infection prevention and control (IPC) policies and inadequate water, sanitation and hygiene (WASH) services stand out [2,3]. Compared to high-income countries, the prevalence of healthcare-associated infections (HAI) in LMIC is high, affecting up to 15% of patients [4,5,6].

Inanimate surfaces and medical/household items (“fomites”) have been identified as reservoirs of pathogens, particularly Gram-negative bacteria associated with outbreaks of HAI [7]. Among these fomites are antiseptics, disinfectants, and hand hygiene products (AS, DI, and HH products) [8,9]. For many decades, in high-income countries these products have been described as reservoirs of multidrug-resistant bacteria. These multidrug-resistant bacteria are nearly exclusively Gram-negative bacteria, and preventive measures to avoid their contamination have been installed [8,10,11,12,13]. In LMIC, the problem and its contributing factors are underreported, particularly in francophone countries from sub-Saharan Africa [9].

The primary objectives of this study were to assess, in AS, DI, and HH products in-use in two tertiary care hospitals in West Africa, (i) the presence of Gram-negative bacteria, (ii) their antimicrobial susceptibility profile, (iii) the associated total bacterial count, and the (iv) factors conducive to bacterial contamination. The secondary objectives were to assess the presence of Gram-negative bacteria in stock and distribution containers.

## 2. Materials and Methods

### 2.1. Definitions of Terms Used in This Study

Antiseptics and disinfectants inactivate microorganisms or inhibit their growth. They are applied on skin or mucous membranes (antiseptics) or on fomites (disinfectants) [3,8,14,15,16]. Unlike antiseptics and disinfectants, soaps have a cleaning action because of their detergent composition. Soaps can be supplemented with antimicrobial agents (medicated soap, antiseptic soap) or not (nonmedicated soap or plain soap) [3,8]. Alcohol-based handrub (ABHR) used for hand hygiene is composed of ethanol or isopropyl alcohol as the active ingredient [3,8]. The different types, functioning and status of product’s containers, and the type of product contamination are defined in Box 1.

### 2.2. Study Design, Setting and Period

This study was conducted in two tertiary care university hospitals, Centre Hospitalier Universitaire Yalgado Ouédraogo (CHU-YO) in Ouagadougou, Burkina Faso, and Centre National Hospitalier Universitaire Hubert Koutoukou Maga (CNHU-HKM) in Cotonou, Benin. Both countries are located in the West Africa region. Burkina Faso is a low-income country, and Benin recently (2020) shifted from a low-income country to the category of lower-middle-income countries [17].

CHU-YO and CNHU-HKM have 697 and 659 beds, respectively, and offer the major clinical sub-specialism services. The wards included the main clinical wards (neonatology, pediatrics, internal medicine, emergency surgery, and maternity). Antiseptics, disinfectants, and hand hygiene products were sampled in a cross-sectional way, from August to November 2019 in CHU-YO and during December 2019 in CNHU-HKM. Hospital management and ward supervisors of both hospitals were informed about the study objectives and methods before the start of the study. To ensure non-biased sampling and interviews, ward staff were not informed about the exact date of sampling.

### 2.3. Ward and Pharmacy Visits, Actual Use and Life Cycle of the Products 

To understand actual use, procedures, and practices related to AS, DI, and HH, ward and pharmacy visits were organized. Observations and interviews were based on a guiding checklist (Supplementary Document S1) and oriented towards the risk factors for contamination along the products’ life cycle in the hospital, as described previously [8]. Every morning the principal investigator (PL) visited the ward, recorded observations, did the interviews and sampled the products. Room temperature and relative humidity were recorded (EBI-20TH1 Xylem Analytics Germany Sales GmbH & Co. KG, Ingolstadt, Germany) and the storage conditions of the AS, DI, and HH products were observed.

Healthcare staff were invited to show the in-use AS, DI, and HH products and to be interviewed. The actual use (indications and applications) of each product was surveyed, as well as the procedures and practices of container reprocessing and filling (topping-up or refilling (Box 1)) [8,9]. In addition, product provider and in-hospital manufacturing, preparation, and distribution practices were traced.

Information recorded for the containers comprised of volume and appearance (opaque, transparent, clean, or dirty), presence and type of dispenser’s system or cap, status (original or aliquoted, reused or recycled (for definitions see Box 1)), and color and homogeneity of the liquid content. In addition, the presence of a glued or written label and its information (product name, brand, concentration, preparation/expiry date, day of opening (first use)) were inspected. For bar soaps, the type of soap box or rack (e.g., perforated plate or grid to allow draining of water excess) and humidity of the surface were recorded.

Further, stock containers (procured branded products) and distribution containers (used to distribute products prepared at the hospital pharmacy and/or products procured as bulk products, see Box 1) were inspected and sampled. If available, sealed stock containers were sampled to investigate intrinsic contamination (i.e., contamination during manufacturing). At both hospitals, containers were photographed. Products in internal medicine, maternity, neonatology, and pediatric wards were sampled in both hospitals. In CHU-YO, additional wards included dialysis, surgery, dermatology-venerology, and odontology-stomatology (Table 1).

### 2.4. Sampling and Transportation to the Laboratory

All the liquid products present in the ward were sampled twice with a sterile single-use pipet. First, 3 mL of surface liquid was sampled in order to optimize the growth of *Burkholderia* spp. [18]. Next, the fluid was gently shaken and 3 mL of the homogenized liquid was sampled. Both samples were transferred in a single-use sterile tube and gently mixed. Bar soap products were collected into a sterile plastic bag using sterile forceps. If too large, the bar was swabbed with a NRS II Transwab (NRS II MW786, Medical Wire & Equipment, Corsham, UK). All samples were kept at 2–8 °C in a cool box with ice packs and transported within 3 h to the laboratory, where they were processed within maximum 4 h after sampling.

### 2.5. Culture Media Inoculation

The liquid samples were vortexed and 100 µL was transferred, using a sterile tip and micropipette on each of three plates (90 mm diameter Petri dish) of well-dried Tryptic Soy Agar (TSA) supplemented with neutralizer (lecithin and polysorbate 80) (Microbial Content Test Agar, Difco, BD Diagnostic systems, Becton Dickinson and Company, Franklin Lakes, NJ, USA). A sterile, disposable L-shaped plate spreader was used to spread the 100 µL on the agar surface. This medium was used for determination of the total colony count (see Section 2.6).

In addition, 100 µL was spread on each of three MacConkey agar No.3 plates (Oxoid, Thermo Fisher Specialty Diagnostics, Basingstoke, Hampshire, UK), i.e., a selective culture medium for non-fastidious Gram-negative bacteria. For bar soap samples, 10 mL of sterile NaCl 0.9% (API Medium, bioMérieux, Marcy l‘Etoile, France) was added to the sampling bag, rubbed for 30 s and next processed as a liquid sample. When the bar soap was too large, a ready to use sterile swab pre-impregnated in neutralizing liquid (Neutralizing Rinse Solution MW786 NRS II Transwab, Medical Wire & Equipment, Corsham Wiltshire, UK) was taken on the exposed surface. After 48 h incubation at 35 °C, each plate was checked for bacterial growth.

Box 1 **Definitions of terms used in this study [8,9,19,20].**

Container: Bottle, vial, or recipient containing a liquid product (antiseptic, disinfectant, or liquid soap).

 In-use container: Container with the product as used by the healthcare workers in the hospital ward.

 Stock container: Large volume (>5 L) container with a branded product as procured and delivered (e.g., liquid soap).

 Sealed container: Stock container not yet been opened at the moment of sample collection.

 Distribution container: Container used to distribute products in the hospital (either branded or in-house prepared products).

Intrinsic contamination: Contamination during manufacturing, evidenced as growth from an original sealed container.

Container design:

 Disposable: Intended and designed for single use.

 Reusable: Intended for reuse (after adequate reprocessing).

Container status:

 Original: Container with a branded product as procured and delivered.

 Aliquoted: Container to which part of the original or in-hospital prepared product was transferred.

 Reused: Repeat use of a container (with or without adequate reprocessing).

 Recycled: Use of a disposable container which was originally used for another product (e.g., alcohol-based hand rub or soft drink).

Reprocessing: Process of cleaning and disinfection between successive use of containers.

Topping-up: Adding product to the container without emptying and reprocessing.

Refilling: Filling the container with product after emptying and adequate reprocessing.

Box or rack: Receptacle of bar soap, it may have a perforated grid or plate to drain water away.

Actual use: Indications and applications of the in-use product as declared by the interviewed healthcare workers (irrespective of the intended use provided by the manufacturer’s instructions or label).

### 2.6. Total Colony Count 

The aerobic colony count (further referred to as “total colony count”) was assessed according to the slightly modified U.S. Food and Drug administration procedure [21,22]. According to this procedure, the optimal number of colonies for counting on a standard 90 mm plate is 25–250 colonies and counts outside these ranges are considered less accurate. In this study, close to 1000 separate colonies were counted with the naked eye on each of the three TSA plates and confluent growth of colonies was considered as >1000 colonies. Next, the average number of colonies per plate was calculated and this average (representing colony counts for 100 µL) was multiplied by 10 to obtain the number of colony forming units per ml (CFU/mL). For the presentation of the data, total colony counts were grouped in 4 intervals, <250; 250–2500; 2501–10,000; and >10,000 CFU/mL.

### 2.7. Identification and Antimicrobial Susceptibility Testing of Bacterial Isolates

At both hospitals, colonies presenting as different morphotypes on MacConkey agar were subcultured, checked for purity, and stored on TSA agar tubes (Difco). Stored isolates were shipped to the Institute of Tropical Medicine and to the University Hospitals of Leuven (UZ Leuven) for identification by Matrix Assisted Laser Desorption Ionization—Time Of Flight (MALDI-TOF) (Bruker MALDI Biotyper, Bruker, Billerica, MA, USA, software version 4.1.80 (PYTH) 102 2017). Isolates for which MALDI-TOF did not provide an acceptable result were identified to the group level of non-fermentative Gram-negative bacteria (NFGNB) by Gram stain reaction and biochemical tests, including oxidase and glucose fermentation on Kligler Iron Agar (Oxoid). Additionally, all *Acinetobacter* isolates were tested for growth at 44 °C in tryptic soy broth to distinguish between *A. baumannii*/*nosocomialis* and the other *Acinetobacter* species [23].

Antimicrobial susceptibility testing was performed for the first isolate of species per sample at the Institute of Tropical Medicine by disc diffusion (Neo-Sensitabs, Rosco Diagnostica, Taastrup, Denmark) according to CLSI guidelines M100-S33 and M45-S31 [24,25]. For the aggregation of antimicrobial resistance data, intermediate susceptibility was grouped together with resistance according to CLSI M39 [26]. Acquired resistance was defined as antibiotic resistance in comparison with the wild-type expected resistance phenotype [27]. Acquired resistance to ≥3 antibiotic classes was considered as multidrug resistance (MDR) [28]. Carbapenem resistance among Enterobacterales, *Pseudomonas,* and *Acinetobacter* species was further assessed for carbapenemase producing enzymes by the RESIST-5 O. K. N. V. rapid immunochromatographic test (Coris BioConcept, Gembloux, Belgium).

### 2.8. Data Entry and Analysis

Data were entered in Excel (Microsoft Office 2019) and analyzed with STATA version 16 (StataCorp, Texas, CA, USA). Proportions (%) were expressed for the quantitative variables when the total number was >10. Differences in proportions were assessed by the chi square test of Pearson or Fisher’s Exact test, and considered significant at a *p*-value < 0.05. After species identification, only the first isolate per species and sample was included for analysis. The results of the cultures were expressed either as proportions of samples or proportion of non-duplicate isolates. Results were primarily presented for both hospitals combined; in case of relevant differences, data for the individual hospitals were presented.

### 2.9. Additional Methods

In addition to the AS, DI, and HH product, eosin 2% water-based solution (a chemical dye) was also retrieved, sampled, and cultured. At the neonatology unit of CHU-YO, it was used for topical treatment of skin and wound care, and two small branded containers (60 mL, Laboratoire GAMET, Ouagadougou, Burkina Faso) were sampled. At CNHU-HKM, eosin 2% was also used for wound care and two samples collected from the preparation room at the pharmacy (aliquoted in recycled containers) were sampled.

In both hospitals, blood cultures were systematically sampled in the scope of routine patient care (BD BACTEC, Becton Dickinson, Franklin Lakes, NJ, USA and bioMérieux, Marcy-L’Etoile, France, respectively). The isolates were sent to the Institute of Tropical Medicine and university hospital of Leuven for identification with conventional techniques and MALDI-TOF confirmation for Enterobacterales and NFGNB, respectively. Blood culture results from the different wards were compiled for a presumptive comparison with the AS, DI, and HH products’ isolates.

As a check for internal quality and potential bias, we assessed design, methods, and results of the present study according to a checklist which we used for a previous systematic review about contaminated AS, DI, and HH products in LMIC [9]. This checklist is based on selected indicators of the Outbreak Reports and Intervention studies Of Nosocomial infection (ORION) and the Microbiology Investigation Criteria for Reporting Objectively (MICRO) guidelines [29,30] and has been adapted to AS, DI, and HH products and the cross-sectional survey method.

## 3. Results

### 3.1. In-Use Products Assessed and Wards Visited, Actual Use of Products

In total, 243 in-use products were sampled (120 in CHU-YO and 123 in CNHU-HKM). They comprised 226 liquid products and 17 bar soap products. Details of products and their actual use are listed in Table 2. Some products were used for several applications. As an example, ethanol 70% was used as antiseptic at both hospitals, but additionally for hand hygiene at CNHU-HKM. Further, Dakin solution—intended as an antiseptic—was also used for environmental cleaning and disinfection, and hand hygiene.

The surveyed inpatient wards represented 70.4% (491/697) and 36.3% (239/659) of beds in CHU-YO and CNHU-HKM, respectively. Most products in both hospitals (73.7%, 179/243 were sampled from hospital risk areas, such as neonatology, pediatrics, maternity, and dialysis (Table 1). Notable differences between both hospitals were the presence of dialysis and surgery (only in CHU-YO) and the size of the neonatology (15 versus 61 beds in CHU-YO versus CNHU-HKM, respectively). The neonatology unit of CNHU-HKM counted 58 samples, as each bed had its dedicated ethanol 70% container for hand hygiene (Table 2). Environmental temperatures recorded during ward visits at CHU-YO ranged between 22.8 and 31.6 °C in August and September, and reached 38.6 °C in October and November; median (range) relative humidity was 60.5% (45.8–82.1%). In CNHU-HKM, temperatures ranged from 26.9 to 32.6 °C; median relative humidity was 73.1% (39.5–80.8%).

### 3.2. Procurement, Preparation and Distribution of Products, Storage in the Wards

**Figure 1:**
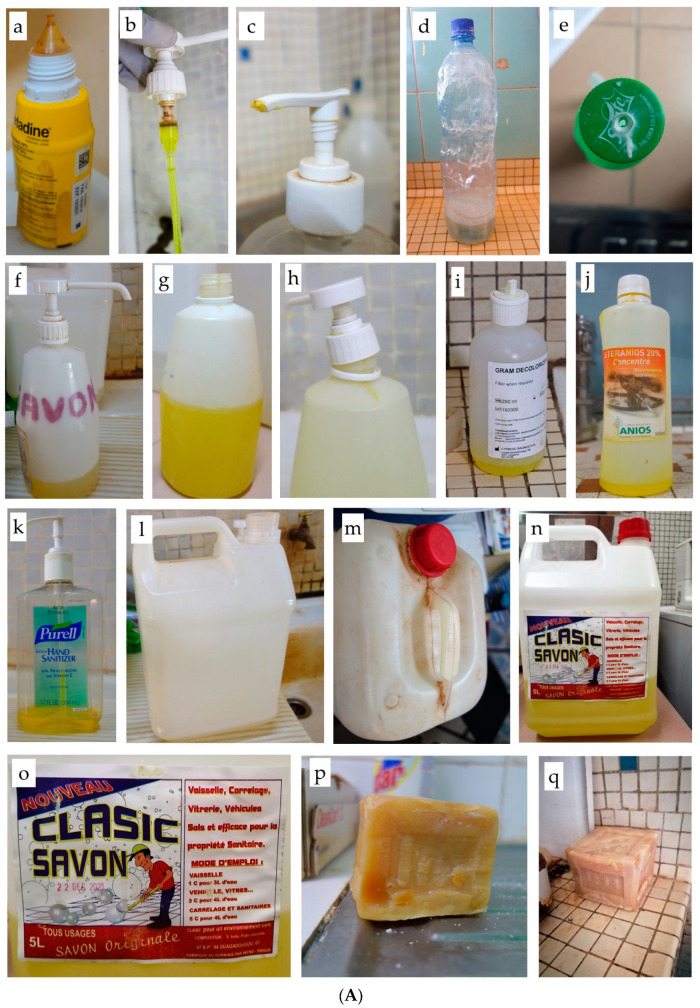

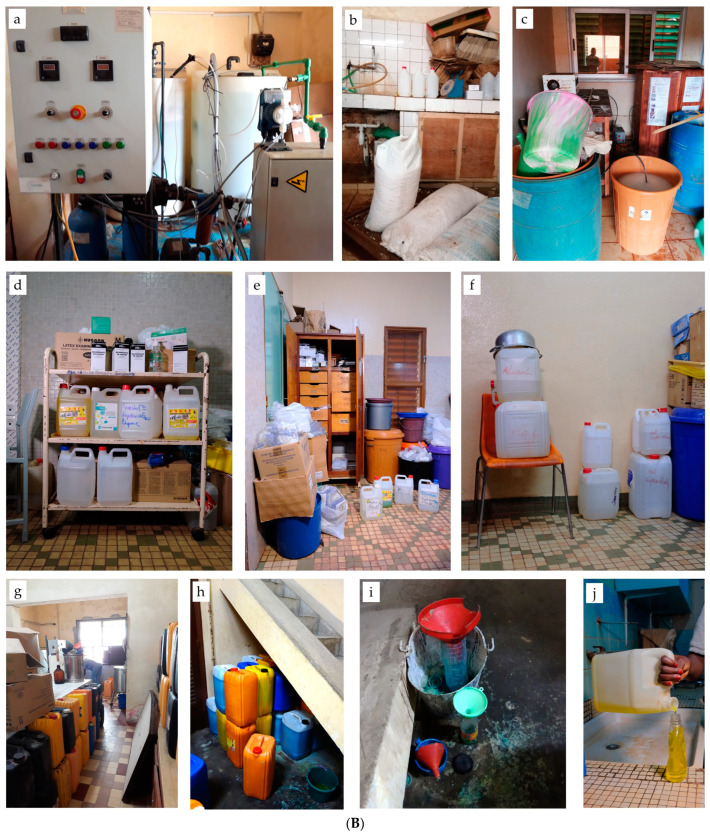
(**A**) Examples of in-use containers at CHU-YO, Ouagadougou, Burkina Faso and CNHU-HKM, Cotonou, Benin. (**a**) Example of container with screw cap and dropper nozzle: povidone iodine in original branded container with complete manufacturer label. (**b**) Overused pump dispenser with brown debris in the pump cylinder. (**c**) Detail of visibly dirty pump dispenser, opening blocked with dried soap residues. (**d**,**e**) Recycled soft drink bottles containing liquid soap: (**d**) Soap, originally colored blue, diluted on-site with tap water in 1.5 L bottle, leading to the grey and foamy aspect. (**e**) Perforated screw cap. (**f**–**h**) Recycled branded hand scrub table-top containers used for liquid soap: (**f**) Handwritten label limited to name of the product. (**g**) Pump dispenser removed, no cap and no label. (**h**) Pump dispenser screw is damaged and does not close tightly. (**i**–**k**) Recycled containers filled with liquid soap, non-labelled, with original (incorrect) label still in place: (**i**) Gram-stain reagent container. (**j**) Disinfectant container. (**k**) Table-top handrub container with pump dispenser, visibly dirty. (**l**–**o**) Intermediate stock and distribution containers: (**l**) Transparent chlorine container, no cap. (**m**) Dirty container containing ethanol. (**n**) Household environmental cleaning soap used for hand hygiene. (**o**) Label detail of household environmental cleaning soap. (**p**,**q**) Bar soap without box (on the bench) and used for hand hygiene in its distributed format (whole large bar). (**B**) Pictures of preparation, storage and distribution of antiseptics, disinfectant, and hand hygiene products in the pharmacy and central storage at CHU-YO, Ouagadougou, Burkina Faso and CNHU-HKM, Cotonou, Benin. (**a**–**c**) Chlorine preparation by sodium chloride electrolysis in dusty and cluttered pharmacy space: (**a**) Sodium chloride electrolysis at CNHU-HKM. (**b**) stock of salt for electrolysis in CHU-YO. (**c**) Sodium chloride electrolysis at CHU-YO. (**d**–**h**) Storage at the ward: cluttered space with stock of antiseptics, disinfectants and hand hygiene products. (**d**,**e**) Ward stock in the neonatology ward of CHU-YO, with handwritten label and original (incorrect) label still in place. (**f**) Stock containers of 70% ethanol, produced in the pharmacy of CHU-YO, with handwritten label. (**g**) Stock containers of ethanol 70% at pharmacy of CNHU-HKM. (**h**) Hospital central storage of liquid soap manufactured by private persons at home and sold in large volume (20 to 30 L) recycled vegetable oil containers. (**i**) Bucket and funnels used to aliquot soap in central storage of CNHU-HKM. (**j**) Topping-up of liquid soap in the neonatology ward of CHU-YO.

Ethanol products (used both as antiseptic and/or hand hygiene) were prepared by the hospital’s pharmacy; a 96% stock solution procured from the national office for essential pharmaceutical products (CAMEG or CAME in Burkina Faso and Benin, at a price of 1245 West African CFA franc (CFA) and CFA 1200 (EUR 1.9 and EUR 1.8) per liter, respectively) was diluted to a working solution of 70% in 200 L barrels. Each hospital pharmacy had a dedicated preparation room, but the preparation rooms were dirty and cluttered with raw materials (Figure 1B).

At CHU-YO, preparation was performed biweekly by the pharmacist; distilled water was used and an alcohol meter was used to check the final concentration. No written procedure was available. At CNHU-HKM, auxiliary staff (i.e., trained in-service but not qualified as pharmacist or pharmacist assistant) prepared the ethanol 70% weekly. As the water distiller was broken, tap water was used. A handwritten procedure was displayed on the wall; it had no date nor version number. Alcohol concentration was not controlled. In both hospitals, ward staff came to the pharmacy to refill their 5–20 L distribution containers when the stock in the ward was nearly finished. Filling was completed with funnels (CHU-YO) or via a tap dispensing container (CNHU-HKM). In the ward, healthcare staff (re)-filled the in-use containers from the distribution containers (see below).

Chlorine 0.5% (sodium hypochlorite) was mostly prepared at the hospital pharmacies by electrolysis (Maxi-WATA^®^, Antenna Foundation, Geneva, Switzerland) of regular salt at a final concentration of 0.5% chlorine, without stabilizer. At CHU-YO, the pharmacist prepared the solution twice weekly. A printed procedure was displayed on the door of a wooden cabinet; it had no date nor version number. Tap water was used for dilution and the quality of the prepared product was verified by a colorimetric titration assay. At CNHU-HKM, the auxiliary staff prepared the chlorine 0.5% daily. Tap water was used. A handwritten procedure was available. The concentration of the final product was not assessed. At both hospitals, ward staff came to the pharmacy to refill their distribution containers (7.8–20 L) once or twice a week; filling was completed with a bucket.

Dakin solution (a stabilized water-based solution of sodium hypochlorite 0.5%; n = 16, all in aliquoted containers) was used in CNHU-HKM only. It was prepared at the pharmacy by auxiliary staff; a handwritten procedure was available. In addition, a single iodine tincture (i.e., alcohol-based iodine) was prepared at the CNHU-HKM pharmacy.

In both hospitals, povidone iodine products (all in-use) were bought by the individual patient at the hospital or private pharmacies as part of a personal medical care or surgical kit.

The remaining antiseptics and disinfectants (chlorhexidine (n = 3, all water-based 4%)), a quaternary ammonium compound (didecyldimethylammonium chloride 0.05%), PAPB (polyaminopropyl biguanide, a chlorhexidine-based product) and eight ABHR samples were branded products procured and distributed by the hospital IPC service.

At CHU-YO, liquid soap was procured and distributed by the hospital IPC service. The product used for hand hygiene was a branded product (Classic Savon, Société Wend Panga, Ouagadougou, Burkina Faso) intended for household environmental cleaning (dish wash, tiles, cars) and delivered in 5 L containers, at a price of CFA 1000 (EUR 1.5) per liter). Ingredients of the liquid soap in CHU-YO were only partly mentioned on the label (“iodine salt and white paste”) and an expiry date was printed on the original container. At CNHU-HKM, liquid soap was procured by the hospital central storage from private persons manufacturing artisanal soap at home and selling it in large volumes (20 to 30 L) containers, at a price of CFA 1580 (EUR 2.4) per liter. For the product in use during the sampling, no information about composition was available and no expiry date was visible.

All but one bar soap product were sampled in CHU-YO. They had been procured by the ward staff and were household-grade plain soap products. The unique bar soap sampled at CNHU-HKM was a private bar soap of a healthcare worker kept in a plastic bag. In-use bar soap products were mostly stored on the bench and without a receptacle (n = 11/16). The remaining five bars were stored in boxes, of which four had a perforated bottom. Six (37.5%) products were visibly wet at sampling.

At the wards, stock and distribution containers for all products except chlorine 0.5% were stored at the ward supervisor’s office. Except for neonatology at CNHU-HKM, there was no dedicated storage place and products were mostly stored on a desk or table in the ward supervisor’s office. None of the products were observed as exposed to direct sunlight. The chlorine 0.5% distribution containers were stored at the housekeeping’s office.

### 3.3. In-Use Containers: Type and Volumes, Status, Labeling, Practices of Reprocessing and Filling

Overall, the 226 liquid products comprised 47 (20.8%) branded products in their original containers and 179 (79.2%) aliquoted containers. Proportions of aliquoted containers were slightly higher in CNHU-HKM compared to CHU-YO (82.0% versus 76.0%, respectively). Table 3 shows the breakdown of container types for the aliquoted liquid soap, Figure 1A,B displays photos from relevant observation of the containers.

More than two-thirds (32/47, 68.1%) of in-use original containers were branded povidone iodine containers with small volumes (125–200 mL) and a screw cap dropper nozzle. In addition, there were eight ABHR products of which six had pump dispensers. Nearly a quarter of original containers (21.3%, 10/47, eight from CHU-YO) were dirty, i.e., the container’s surface was dusty and/or contained product’s residues. The manufacturer’s label was present on 44/47 original containers. In addition to product name, it listed the products’ concentration and expiry date, except for four and three labels, respectively. Five products (three povidone iodine and one of chlorhexidine and ABHR) were expired at the moment of sampling. None of the original containers had a date of first use written on them.

Liquid soap, ethanol 70%, Dakin and chlorine 0.5% solutions were nearly exclusively distributed in aliquoted containers and also represented the majority (172/179, 96.1%) of aliquoted containers. Nearly all (95.5%, 171/179 of the aliquoted containers, representing 75.6% (171/226) of the in-use liquid containers) were recycled containers, most of them (48.5%, 83/171) were table-top branded surgical hand wash or ABHR containers designed and marketed as single-use. In addition, there were 16.4% (28/171) polyethylene terephthalate (PET) bottles originally used for soft drinks. Most of the aliquoted containers (91.1%, 163/179) had volumes exceeding 300 mL. Recycled containers were indefinitely re-used. None of the containers showed inhomogeneous content.

At CNHU-HKM, all table-top containers with a pump dispenser (n = 46) were in good condition, as they had recently been recycled from a collection of AHBR containers distributed as part of a hospital-wide hand hygiene program. In part, they explained the lower proportion of dirty containers in CNHU-HKM compared to CHU-YO (Table 3). In CHU-YO, three table-top dispensing containers had a broken pump, and in four containers the pump dispensing system had been removed. In-use aliquoted chlorine products (n = 12) were stored in washing basins or recycled detergent containers; some had no cap nor lid and three were transparent.

Only 8/179 (4.5%) of aliquoted containers were labeled; all observed in CNHU-HKM and all hand-written with an indelible marker. The information on the label was limited to the name of the product; one label mentioned the concentration. On 94/179 (52.5%) of the recycled containers the original product label (e.g., ABHR) was still in place. The date of first use was written on none of the aliquoted containers.

### 3.4. Distribution, Stock and Sealed Containers

A total of 16 stock containers (all at CHU-YO) were retrieved, all were dirty (Supplementary Table S1). Two products (liquid soap and chlorhexidine) were expired. The date of opening was not recorded on the container, the period-after-opening was not defined. Five containers were still sealed and were opened on site for sampling. Further, 41 distribution containers were sampled. They consisted of 5 to 25 L screw-cap containers which were either reused generic containers or recycled containers which had originally been used for vegetable oil, soap, or dialysis fluid. All but seven were dirty and none was appropriately labeled. The original product label (e.g., dialysis fluid) was still in place for some products (n = 4).

### 3.5. Procedures and Practices for In-Use Products

Neither in CHU-YO nor CNHU-HKM were hospital-validated procedures about hand hygiene or the use of antiseptics and disinfectants available. None of the hospitals had a hospital-approved product procurement list or a quality management system to orient and monitor the selection of products, formulation, concentration, and supplier. Registration of production and distribution made by the pharmacy, IPC service, central procurement, and the wards was limited to the product name and quantities.

Likewise, there were no procedures for the selection, reprocessing and refilling of containers. Except for the initial use of recycled containers (i.e., removing of remnants of the original product and subsequent rinsing), 78.8% (141/179) of aliquoted containers had not been reprocessed. Further, 77.6% (139/179) of aliquoted containers were filled by topping-up from the distribution container, which in turn was refilled at the pharmacy or IPC service without reprocessing. It is of note that, in the neonatology wards in CNHU-HKM, the in-use containers (n = 35) were cleaned daily. However, before cleaning, the content of the container was decanted into another container, and afterwards the content was poured back into the cleaned container.

In both hospitals more than a third of in-use liquid soap products (37.8% (14/37) and 37.5% (12/32) in CHU-YO and CNHU-HKM, respectively) had been further diluted by the ward staff with tap water according to their estimate of the ideal viscosity. An incidental observation in the neonatology ward at CHU-YO were cotton balls soaked in ethanol 70% used for the disinfection of thermometers.

### 3.6. Growth of Gram-Negative Bacteria from In-Use Products, Association with Total Bacterial Counts

Overall, 179 suspected Gram-negative bacterial isolates from in-use products were sent from the study sites (101 from CHU-YO and 78 from CNHU-HKM). Upon subculture, 17 isolates did not grow. After removing of 56 duplicate isolates, a total of 105 single isolates were obtained; 88 were recovered from liquid soap and the remainder from the other products.

**Figure 2:**
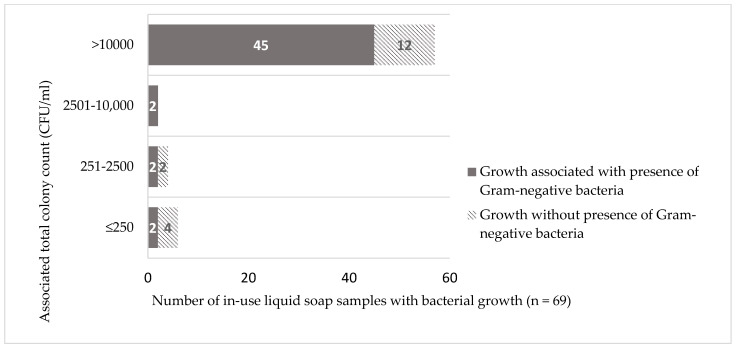
Growth of Gram-negative bacteria with associated total colony counts of in-use liquid soap products in CHU-YO, Ouagadougou, Burkina Faso and CNHU-HKM, Cotonou, Benin. Abbreviation: CFU/mL = colony forming unit per milliliter. Among the 45 samples with presence of Gram-negative bacteria and associated colony counts >10,000 CFU/mL, 41 had confluent colony growth on MacConkey agar.

Nearly three-quarters (51/69, 73.9%) of liquid soap samples grew with Gram-negative bacteria (Table 2). Contamination occurred consistently across different wards (Table 1). Proportions were higher in CNHU-HKM compared to CHU-YO (84.4% (27/32) versus 64.9% (24/37), respectively), but the difference did not reach statistical significance (*p* = 0.6). Out of these 51 samples, 45 (88.2%) were associated with total colony counts exceeding 10,000 CFU/mL in which category they represented the largest share (45/57 samples, 78.9%), (Figure 2).

Out of 88 isolates from liquid soap, there were 14 isolates belonging to the Enterobacterales (*Enterobacter* spp. n = 4, and *Klebsiella* spp. n = 6) or the *Aeromonas/Vibrio* non-cholerae groups (n = 4), as well as 14 isolates of *Pseudomonas aeruginosa* (Table 4). All but 2 of these 28 isolates were associated with total colony counts >10,000 CFU/mL. They were obtained from CHU-YO (n = 8 isolates), as well as from CNHU-HKM (n = 20), concentrated in the neonatology and maternity wards (17 and 3 isolates, respectively). The remaining 60 isolates were NFGNB comprising *Pseudomonas* spp. (n = 21), *Acinetobacter* spp. (n = 6) and other NFGNB (n = 33), of which 49 were associated with total colony counts >10,000 CFU/mL. Among the 60 remaining NFGNB, 28 did not meet criteria for acceptable species identification by MALDI-TOF.

In 4/17 bar soap samples (all obtained in CHU-YO), seven Gram-negative species were grown. They included Enterobacterales (n = 4) and NFGNB, all but one associated with total colony counts >10,000 CFU/mL (Table 5).

The remaining products with growth of Gram-negative bacteria were chlorhexidine (1/3 samples), QUAT (1/2 samples), chlorine (2/12 samples), ABHR (2/11 samples) and ethanol 70% (2/80 samples) (Table 1); apart from one chlorhexidine product, they were obtained in CHU-YO. They yielded 11 isolates, all NFGNB and—except for the ethanol 70%—associated with high colony counts. The two contaminated samples of ABHR that grew *P. aeruginosa* were obtained from recycled non-labeled containers (Table 5). No growth of Gram-negative bacteria was observed from the samples with povidone iodine and Dakin solution (Table 1).

### 3.7. Growth of Gram-Negative Bacteria from Stock and Distribution Containers

Liquid soap distribution and in-use stock containers were the most frequently contaminated with Gram-negative bacteria, they represented 10/17 containers, all but one had total colony counts >10,000 CFU/mL. (Supplementary Table S1). One of these containers (in CNHU-HKM neonatology) grew *Enterobacter cloacae* complex and *Klebsiella* spp., the others grew NFGNB. Two sealed soap containers did not grow bacteria. Among the ethanol 70% distribution containers, 2 out of 21 grew Gram-negative bacteria.

### 3.8. Antimicrobial Resistance of Gram-Negative Bacterial Species

MDR was found among 9/16 (56.3%) of the Enterobacterales, all of which had extended-spectrum-beta lactamase (ESBL) production (Supplementary Table S2). The involved bacteria that were resistant were *Klebsiella pneumoniae* (n = 5), *Klebsiella oxytoca* (n = 2), and *Enterobacter cloacae* (n = 2), isolated in liquid soap used at CNHU-HKM (neonatology (n = 6) and maternity (n = 1)), and in bar soap used at CHU-YO surgery (n = 2). Among the NFGNB, MDR was found in three *Acinetobacter* isolates, including *A. venetianus* (CNHU-HKM maternity), *A. baumannii* (CHU-YO pediatrics), and *A. indicus* (CHU-YO dialysis) (Supplementary Table S3). In addition, the MDR *A. baumannii* from chlorine in the pediatric ward was meropenem resistant and produced New Delhi metallo-beta-lactamase (NDM). Further, a *Pseudomonas stutzeri* group isolate from liquid soap in CHU-YO pediatric ward was found resistant to meropenem, and tested positive for Verona Integron-encoded metallo-beta-lactamase (VIM group) carbapenemase.

### 3.9. Additional Results

The eosin 2% solutions in-use at CHU-YO neonatology grew (*Klebsiella pneumoniae* (n = 1), *Pseudomonas boreopolis* (n = 1) in one sample, and *Pseudomonas aeruginosa* (n = 1) and *Cupriavidus pauculus* (n = 1)) in the second sample, associated total colony counts >10,000 CFU/mL. The sample at CNHU-HKM (sampled at the pharmacy) did not grow Gram-negative bacteria.

Supplementary Tables S4 and S5 list the bacterial species isolated from blood cultures at CHU-YO and CNHU-HKM according to ward for the period January 2019—January 2020 and July 2019—June 2020, respectively, i.e., 6 months before and after the sampling period. The species distribution reflected a high proportion of potentially healthcare-associated pathogens (*Klebsiella* spp., *Enterobacter* spp., NFGNB accounting for 85/117 (72.6%) and 138/174 (79.3%) of isolates at CHU-YO and CNHU-YKM, respectively. Species identities overlapping with those from contaminated AS, DI, and HH products were *Klebsiella pneumoniae*, *Acinetobacter* spp., *Pseudomonas putida,* and *Pseudomonas stutzeri*. Supplementary Table S6 displays the color-coded results for the study quality and risk of bias checklist. Most indicators were scored as “good”. Items scored as “satisfactory” were product ingredients (not retrievable for liquid soap products), actual use of products (multiple and off-label use of products), and relatively low number of antiseptics and disinfectants, stock and distribution containers.

## 4. Discussion

### 4.1. Main Findings

In two tertiary care hospitals in West Africa, the growth of Gram-negative bacteria was demonstrated in a quarter (25.5%, 62/243) of AS, DI, and HH products, ranging from absent or rare in alcohol-based products, povidone iodine, and Dakin solution to nearly three-quarters (73.9%) in liquid soap products. Contaminating flora included Enterobacterales and the *Vibrio* non-cholerae/*Aeromonas* group, as well as NFGNB (20.6% and 79.4% among a total of 105 isolates, respectively). The presence of Gram-negative bacteria was associated with high total bacterial counts (>10,000 CFU/mL). MDR was present among 9/16 *Enterobacterales* and 3/14 *Acinetobacter* spp., including carbapenem resistance. Along the life cycle of the products, multiple factors associated with bacterial contamination were noted and contamination was demonstrated in stock and distribution containers.

### 4.2. Comparison with Previous Findings: Proportion among Products, Bacterial Species

Healthcare-associated outbreak reports and cross-sectional studies demonstrating contaminated antiseptics and disinfectants have been published for over 50 years, whereas contaminated liquid soap products have been mostly published since the 2000s [8]. A total of 15 hospital outbreaks associated with liquid soap have been reported (all but 2 since 2000), of which 11 [31,32,33,34,35,36,37,38,39,40,41] and 4 [42,43,44,45] were in HIC and LMIC, respectively; the vast majority was caused by Gram-negative bacteria. As for cross-sectional surveys, 8/25 surveys in LMIC had found contaminated liquid soap samples, all published after 2000 [9].

In the present study, the proportion of liquid soap samples with growth of Gram-negative bacteria (64.8% and 87.1% in CHU-YO and CNHU-HKM, respectively) stood out compared to the other products. It was also considerably higher than previously reported from LMIC which was—when intrinsic contamination and low counts of staphylococci were subtracted—between 0 and 17.1% [9]. The reasons for the presently high contamination ratio are probably an interplay of multiple factors conducive to contamination (see below) in a setting with fewer resources compared to the previously published articles which originated from higher income level countries in Southern and Western Asia [46,47,48,49,50,51].

In addition, the contaminating flora of liquid soap included virulent organisms, such as *Klebsiella* spp., *Enterobacter* spp., *Aeromonas* spp., *Vibrio alginolyticus* (which were exclusively present in soap products), and *P. aeruginosa* (n = 16), associated with high total bacterial counts (>10,000 CFU/mL) and present in high-risk areas (neonatology, maternity). The association of liquid soap products with Enterobacterales (*Serratia* spp., *Klebsiella* spp., and *Enterobacter* spp.) and high total colony counts was reported in previous surveys and outbreak reports from both HIC and LMIC [8,9]. A potential explanation for this association is the ability of Enterobacterales to colonize the healthcare workers’ hands [52,53] which, subsequently, can cause retrograde contamination of containers during handwashing (e.g., by touching the spout) or topping-up [32,39,54].

About a quarter of bar soap samples (4/17, 23.5%) were contaminated with Gram-negative bacteria and this proportion was considerably lower compared to liquid soap products. It was also lower than the >50% proportions reported in most previous publications [48,50,51,55]; in part, this difference may be due to a lower availability and a more selective use of bar soap at the present study sites. Most studies comparing side-to-side bar soap with liquid soap found that bar soap was the most frequently contaminated [8,9]. Species distributions and the high associated total colony counts in bar soap were similar to those in liquid soap products, in line with previous reports [50,52,55,56].

Other products grown with Gram-negative bacteria were chlorhexidine, QUAT, and chlorine; species were NFGNB associated with high colony counts. Water-based antiseptics and disinfectants are known for their vulnerability to contamination with Gram-negative bacteria and contaminated products may reach high colony counts, particularly if over-diluted [8,9]. By contrast, alcohol-based products are typically insensitive to bacterial contamination, but may be contaminated with spore forming organisms, such as *Bacillus* spp. [57,58]. In the present study, only 2/80 ethanol 70% grew with Gram-negative bacteria but associated colony counts were less than 500 CFU/mL; both products were collected from 1 L unlabeled aliquoted containers. Further, *P. aeruginosa* grew from two ABHR samples at colony counts >10,000 CFU/mL, which was an unexpected finding. The products were however stored in aliquoted non-labeled containers, and factors, such as biofilm and a too low product concentration, might have facilitated this contamination.

None of the samples with povidone iodine and Dakin solution grew with Gram-negative bacteria. Dakin solution is not widely used and only a single healthcare-associated outbreak related to this product has been reported [59]. The reasons for the apparent absence of contamination despite its distribution in aliquoted containers may be the relatively high and stable product concentration (available chlorine 0.5%). Iodophor-based products (such as povidone iodine) are water-based too and are susceptible to contamination with Gram-negative bacteria [8,60]. In the present setting, the absence of contamination may be explained by the fact that nearly all povidone iodine samples were branded products in low volume containers procured and used by individual patients.

### 4.3. Multidrug Resistance

Over half of the isolated Enterobacterales were MDR (including resistant to third generation cephalosporins) and, hence, listed as of critical priority on the WHO list of pathogens for which research and development of new antibiotics is required [61]. This finding confirms those from previous outbreak reports and surveys of AS, DI, and HH products in LMIC [9] and is in line with the high proportions of MDR in a blood culture surveillance study in a referral hospital in rural Benin [62].

The proportion of MDR in the current survey was lower among the NFGNB, but given their natural resistance against multiple antibiotic classes and the limited access to reserve antibiotics and therapeutic drug monitoring, infections caused by these species are considered as “difficult-to-treat” in LMIC [62,63]. Of particular concern are the carbapenemase enzymes among *Acinetobacter* spp. and *P. stutzeri*, respectively. Both NDM and VIM belong to the class A or metallo-beta-lactamases, which are not susceptible to the newly developed beta-lactamase inhibitors. This leaves few therapeutic options [64]. Carbapenem-resistant *Acinetobacter baumannii* is listed as a critical pathogen too [61]; *P. stutzeri* is an upcoming opportunistic pathogen and its VIM-2 production has been described from different parts in the world [65].

### 4.4. Potential Causes Explaining for the High Proportion of Contaminated Products

Although the present study was not designed to trace causes of contamination, several factors known to promote bacterial contamination of AS, DI, and HH products [8,9] were observed along the products’ life cycles in the hospital.

#### 4.4.1. Product-Related Factors: Ingredients, Manufacturing, In-Hospital Preparation

Our limited search did not reveal intrinsic contamination of delivered products, but ingredients and expiry date of the liquid and bar soap samples were unknown in both hospitals. Given the small-scale and in-house manufacturing of the soap products, it may be possible that preservatives (agents that destroy or inhibit growth of microorganisms) were lacking or that non-sterile water was used.

Likewise, potential risk factors were observed at the in-hospital preparation of products, such as the absence of procedures, preparation by auxiliary staff, cluttered and ill-organized working spaces, the use of tap water instead of distilled or freshly boiled water, the use of non-sterile items for aliquoting, and (in at least one site) the lack of tools for verification of alcohol and chlorine concentration [8,9].

#### 4.4.2. Containers

Three-quarters of in-use containers were recycled disposable containers or soft drink bottles, some of which were partially broken and overused and most had large (>300 mL) volumes. They were not reprocessed but re-used until they were completely unfit for use; broken or removed lids and dispensers were observed. Labeling was largely insufficient, in particular for aliquoted containers; less than 5% were labeled, >50% of recycled containers still had the label of the original product affixed, none had expiry date or day-of-opening labeled. The situation was similar for stock and distribution containers.

Access to appropriate, well-functioning, and biosafety-proven containers is a serious problem in LMIC [66] and malfunctioning containers are barriers to proper hand hygiene practices [66,67]. Most disposable containers are made of high-density polyethylene which is not autoclavable [9]. In addition, the pump cylinder of table-top containers and the atomizer (spray-unit) of spray bottles are inaccessible for mechanical cleaning and disinfection [8]. The re-use of pump cylinders ensured the contamination of bulk refillable dispensers in the community and the hospital settings [36,38,39,68,69,70]. Further, dead spaces behind plastic liners of screw caps (as in recycled soft drink bottles) facilitate biofilm formation, which protects bacteria from desiccation but also from antiseptics and disinfectants [71]. Too large volume containers entails prolonged use which, in turn, facilitates biofilm production [19] while preservatives degrade over time [68].

#### 4.4.3. End-User Practices

Other risks for contamination were related end-user practices [9]: expired products were noted among in-use as well as stock products; for the liquid soap product in CNHU-HKM, no expiry date was set. In both hospitals, period-after-opening for in-hospital prepared products was not defined.

Topping-up (practiced in three-quarters of the aliquoted containers) is a well-known and persistent risk practice of contamination, as is the habit of keeping cotton balls impregnated in antiseptic [8,9]. The practice of diluting liquid soap with tap water up to the desired viscosity was observed in both hospitals and has not been described before. Particularly in rural areas, healthcare facilities in low-resource settings rely on non-piped improved water sources (boreholes, rainwater collection) which may be affected by fecal contamination [2].

#### 4.4.4. Factors behind the Causes of Contamination

Behind the causes mentioned above, there are economic, managerial, and human factors. Economic considerations may have influenced the procurement of liquid soap products of unknown composition and quality. Quality management was weak (procedures, stock management,) and oversight was lacking (fragmented and partly decentralized procurement and supply system). Further, human factors may also interfere and incite incorrect users’ practices [8]; an example was the apparent low risk perception about the use of tap water in the ward, as well as in the pharmacy.

### 4.5. Limitations and Strengths

The cross-sectional design of the study and the absence of documented stock management did not allow the study to trace information about production, expiry dates and the day of first opening. These limitations precluded a formal analysis of the potential risk factors. Further, the sample selection was focused on the in-use products present in the wards, resulting in an underrepresentation of antiseptics and disinfectants. Although we conducted upstream analysis of the in-hospital distributed products, we did not systematically assess intrinsic contamination and content. As discussed above, we could not trace the ingredients of the soap products, hampering full understanding of their high contamination ratios. In addition, only the total colony count was performed and not Gram-negative bacterial colony count. Finally, despite precautions (non-advertised ward visits), it cannot be ruled out that staff of some wards (alerted by colleagues from other wards) would have discarded too old products or too dirty containers. As with any observation or interview, there was a risk of bias towards presumed desirable or correct answers.

As to the strengths, apart from the lack of information about the ingredients of the liquid soap products, the study complied with the risk of bias and quality checklist [9], Supplementary Table S4. It is notable that sampling was systematically and representative for both sites, and culture techniques (sampling techniques, neutralizer, quantitative cultures, and antimicrobial susceptibility testing) were up-to-date.

Further, the cross-sectional design enabled the assessment of side-to-side products from multiple wards and two centers and to conduct interviews and observations in a stress-free context (unlike, for instance, during an outbreak investigation). Moreover, the study was supported by the hospitals’ management and the principal investigator (PL) conducted ward visits and staff interviews in person, building-up trustful relations with the hospital staff. This allowed us to map the lifecycle of the products in the health facility and to appreciate the associated risk factors, as performed previously in other surveys [72,73,74].

### 4.6. Relevance

Among the isolates recovered, *Klebsiella pneumoniae*, *Enterobacter* spp., *Pseudomonas aeruginosa* and *Acinetobacter baumannii*, are leading Gram-negative species of healthcare-associated infections in low- and middle-income countries [75]. These species, as well as other species presently recovered have been previously implicated in healthcare-associated outbreaks related to AS, DI, and HH products [8,9]. In the present survey, they were associated with high total colony counts and MDR, they had overlap with clinical isolates and they were obtained from in-use products of high-risk areas.

As to healthcare-associated infections and outbreaks, different transmission routes are possible. Contaminated antiseptics mostly cause infections through local application during invasive acts (intravascular and urinary catheters, surgery) or topical care (wound, tracheotomy). Disinfectants cause infections via contact with semi-critical (surgical instruments, transfer forceps) or non-critical items (thermometers, septa of multidose vials) [8,9]. In the case of liquid soap, handborne transmission applies, i.e., colonization of healthcare workers’ hands followed by transfer to patients, which, in turn, causes colonization and subsequent infection [42,43,44].

Notable susceptible patients are neonates, given their immature skin and mucosal barriers—outbreaks related to contaminated liquid soap products in neonatal wards have been reported from HIC and LMIC [32,33,38,42,45,76]. In the present study, contamination with Gram-negative bacteria occurred in products across all wards, but liquid soap products heavily contaminated with virulent bacteria (Enterobacterales and *P. aeruginosa*) concentrated in high-risk wards, such as maternity and neonatology. Moreover, contamination even at high colony counts was not visible to the naked eye (no inhomogeneous content, no discoloration, clean containers) and consequently was not perceived; this observation is also in line with previous findings [8,9].

An experimental study showed that handwashing with contaminated liquid soap at a 30 s rinse with good quality water transmitted Enterobacterales at a threshold count of 3700 CFU/mL or higher [77]. In line with this, washing hands with plain liquid soap contaminated with *P. aeruginosa* at 100,000 CFU/mL did not transfer *P. aeruginosa* from soap to hands when abundant rinsing was applied, but did so at a brief rinsing [78]. As only half of health care facilities in sub-Saharan Africa have access to basic water services [2], abundant rinsing with good-quality water will not be possible in many low resource conditions. In the present study, the maximum bacterial counts were capped at 10,000 CFU/mL and Gram-negative counts were not determined. Given the confluent growth on MacConkey agar in most (41/45) liquid soap samples with Gram-negative bacteria and their associated counts higher than 10,000 CFU/mL (Figure 2), it can be expected that their Gram-negative counts were much higher than the above-mentioned threshold of 3700 CFU/mL.

In conclusion, the observed contaminated products are a potential threat to healthcare associated infections. On top of this, in both hospitals, contaminated liquid soap products were used in high-risk wards (such as neonatology) units and were contaminated with clinically relevant Enterobacterales, *Aeromonas*/*Vibrio* non-cholerae, *Pseudomonas aeruginosa*, and *Acinetobacter* spp.

### 4.7. Generalizability

To our knowledge, apart from one outbreak investigation (Senegal, 1987 [59]), the present study is the first one from francophone West Africa to document contamination of AS, DI, and HH products. Despite some differences between the sites, the main findings were similar and are expected to be representative for other low- and middle-income countries, particular in sub-Saharan Africa. However, both sites were urban tertiary care centers equipped with a functional clinical laboratory service. Given the low access to water, sanitation, and hygiene services in healthcare facilities in sub-Saharan Africa and the virtual absence of clinical bacteriology services in many areas [2,79], frequencies and impact of contamination of AS, DI, and HH products in rural areas in sub-Saharan are probably much more frequent but not recognized and consequently underreported.

The present results support the WHO recommendation to prefer ABHR rather than liquid soap for hand hygiene in the healthcare setting (except for a few situations) [3]. This recommendation is based on efficacy studies [80] and can be supported the high vulnerability to contamination of liquid soap products. Although the exact composition of the liquid soap products was unknown, it was household grade and intended for environmental cleaning and most probably “plain soap”, i.e., with no antiseptics added. The question whether antiseptic soap is less prone to bacterial contamination has been noted by WHO more than a decade ago [3], but is still not clear; a cross-sectional survey of soap dispensers in food establishments in the U.S. showed that antiseptic soap products were less contaminated compared to plain soap products [81] but in the healthcare setting, bacterial contamination of liquid soap has been described for both plain and antiseptic soap products [8,9].

The present findings may also apply to related products in healthcare, such as hospital water, handwashing stations, cleaning agents, mouthwash, and other products which are used for topical care [9]. An example in the present study was the contaminated water-based eosin 2% products used for local skin care used in CHU-YO.

### 4.8. Risk Mitigation, Outstanding Issues and Future Research

For risk mitigation of bacterial contamination of AS, DI, and HH products, we refer to the recent “*Best Practices*” and outstanding issues compiled in a recent review of this topic [9]. The present study highlights several of outstanding issues, such as the need of field-adapted reusable containers and period-after-opening adapted to LMIC environmental conditions, and understanding and correcting human factors behind inappropriate practices. In addition, the study reveals the need for minimal product quality criteria for liquid soap products. Meanwhile, since the implementation of best practices requires efforts, resources, and time, a risk-based approach is recommended. An example is the prioritization of ABHR and branded products in their original containers to high-risk hospital wards, such as oncology and neonatology [43,82] or to invasive procedures. Cost of ABHR may be a (perceived) problem, but retail prices of ethanol 96% noted in this study were similar and even lower than those for the liquid soap product in Burkina Faso and Benin, respectively. Further, given most in-use containers were recycled disposable (not autoclavable) containers, a reprocessing system comprising soaking in chlorine and subsequent rinsing with freshly distilled or boiled cold water can be a first approach.

## 5. Conclusions

The present study illustrates the contamination by Gram-negative bacteria of AS, DI, and HH products in two tertiary care hospitals in sub-Saharan Africa. Liquid soap products stood out in frequency of contamination (nearly three-quarters, total bacterial counts mostly >10,000 CFU/mL) and clinically relevant and MDR bacteria (half of isolated Enterobacterales). Multiple and intertwined risk factors along the line of preparation and distribution of products were observed, including on-the-spot dilution with tap water and indefinite re-use of recycled disposable containers. Considering the pivotal role of hand hygiene in preventing transmission of healthcare-associated infections [16], the present findings urge for increased awareness for the potential contamination of liquid soap products, including quality criteria for products, access to field-adapted containers, and implementation of appropriate reprocessing procedures.

## Figures and Tables

**Table 1:** Ward distribution of in-use antiseptics, disinfectants and hand hygiene products sampled at CHU-YO, Ouagadougou, Burkina Faso and at CNHU-HKM, Cotonou, Benin. Numbers represent the number of products sampled in the wards; within brackets are the number of products with growth of Gram-negative bacteria. Abbreviation: AS = antiseptic, HH = hand hygiene, PAPB = polyaminopropyl biguanide, QUAT = quaternary ammonium compound.

Products Collected per Hospital Ward	Ethanol 70% (AS)	Ethanol 70% (HH)	Povidone Iodine	Alcohol-Based Handrub	Chlorhexidine	Dakin	Iodine Tincture	Chlorine	QUAT	PAPB	Liquid Soap	Bar Soap	Total
**CHU-YO**	**31 (2)**	**1**	**14**	**8 (1)**	**1**	**-**	**-**	**10 (2)**	**1 (1)**	**1**	**37 (24)**	**16 (4)**	**120 (34)**
Surgery ^a^	3 (1)	-	2	1 (1)	-	-	-	1	-	-	9 (4)	5 (2)	21 (8)
Internal medicine	3 (1)	-	-	-	-	-	-	1	-	-	6 (6)	3	13 (7)
Maternity	6	1	4	1	-	-	-	-	-	-	6 (4)	-	18 (4)
Neonatology	3	-	1	3	-	-	-	3		1	3 (1)	3 (2)	17 (3)
Dialysis	3	-	-	-	-	-	-	-	1 (1)	-	4 (4)	1	9 (5)
Pediatrics ^b^	7	-	3	2	-	-	-	5 (2)	-	-	6 (5)	3	26 (7)
Others ^c^	6	-	4	1	1	-	-	-	-	-	3	1	16
**CNHU-HKM**	**18**	**30**	**19**	**3**	**2 (1)**	**14**	**1**	**2**	**1**	**-**	**32 (27)**	**1**	**123 (28)**
Internal medicine	3	-	8	-	-	1	-	-	-	-	1 (1)	1	14 (1)
Maternity	7	1	10	1	1 (1)	4	1	-	-	-	7 (6)	-	32 (7)
Neonatology	4	29	1	2	1	2	-	2	1	-	16 (16)	-	58 (16)
Pediatrics ^b^	4	-	-	-	-	7	-	-	-	-	8 (4)	-	19 (4)
**Total**	**49 (2)**	**31**	**33**	**11 (1)**	**3 (1)**	**14**	**1**	**12 (2)**	**2 (1)**	**1**	**69 (51)**	**17 (4)**	**243 (62)**

^a^ including emergency surgery, trauma surgery, anesthesia, and resuscitation room. ^b^ including pediatric emergency, pediatric hospitalization and oncology (at CHU-YO and CNHU-HKM) and pediatric clinic at CHU-YO. ^c^ including dermatology-venerology and odontology-stomatology.

**Table 2:** In-use products; type of containers; procurement, supply and actual use of antiseptics, disinfectants, and hand hygiene products in CHU-YO, Ouagadougou, Burkina-Faso and CNHU-HKM, Cotonou, Benin. The data represent the number of samples. For products contaminated with growth of Gram-negative bacteria, numbers are written between brackets. Abbreviations: AS/DI/HH = antiseptics, disinfectants and hand hygiene products, PAPB = polyaminopropyl biguanide, QUAT = quaternary ammonium compound. Numbers of actual use may outnumber total of products as some products had multiple use.

In-Use Products	Used as	Samples			Containers		Procurement and Supply	
		CHU-YO	CNHU-HKM	Total	Aliquoted	Original		
Antiseptics/disinfectants *		67 (6)	90 (1)	157 (7)	110	47		
Ethanol 70%	AS/DI /HH	32 (2)	48	80 (2)	79	1	CHU-YO:	prepared by the hospital pharmacy (n = 32) branded product (n = 1)
							CNHU-HKM:	prepared by the hospital pharmacy (n = 48)
Povidone iodine 4% and 10%	AS	14	19	33	1	32	CHU-YO:	branded product (n = 13) aliquoted product, origin not traceable (n = 1)
							CNHU-HKM:	branded product (n = 19)
Dakin	AS/DI	-	14	14	14	-	CNHU-HKM:	prepared by the hospital pharmacy (n = 14)
Chlorhexidine 4%	AS	1	2 (1)	3 (1)	1	2	CHU-YO:	branded product (n = 1)
							CNHU-HKM:	branded product (n = 1) branded product aliquoted in the ward (n = 1)
Iodine tincture	AS	-	1	1	1	-	CNHU-HKM:	prepared by the hospital pharmacy (n = 1)
Chlorine 0.5%	DI	10 (2)	2	12 (2)	10	2	CHU-YO:	branded product (n = 2) prepared by the hospital pharmacy (n = 10)
							CNHU-HKM:	prepared by the hospital pharmacy (n = 2)
QUAT	DI	1 (1)	1	2 (1)	1	1	CHU-YO:	branded product (n = 1)
							CNHU-HKM:	branded product aliquoted in the ward (n = 1)
PAPB	DI	1	-	1	-	1	CHU-YO:	branded product, polyaminopropyl biguanide 0.36% (n = 1)
Alcohol-based hand rub	HH	8 (1)	3	11 (1)	3	8	CHU-YO:	branded products (n = 6) aliquoted products, origin not traceable (n = 3)
							CNHU-HKM:	branded products (n = 2) aliquoted products, origin not traceable (n = 1)
Soap products		53 (28)	33 (27)	86 (55)	69	-		
Liquid soap *	HH	37 (24)	32 (27)	69 (51)	69	-	CHU-YO:	branded products (5 L containers), stored and aliquoted in the ward (n = 37)
							CNHU-HKM:	artisanal small scale manufactured by private person (20–25 L containers) (n = 32)
Bar soap	HH	16 (4)	1	17 (4)	NA	NA	CHU-YO:	branded products, household grade soap (n = 12) home-made product, no brand name (n = 4)
							CNHU-HKM:	home-made product, no brand name (n = 1)
Total		120 (34)	123 (28)	243 (62)	179	47	-	-

* Contamination rate of antiseptics/disinfectants (4.5%, 7/157) versus liquid soap (73.9%, 51/69) *p* < 0.0001.

**Table 3:** Overview of the recycled in-use liquid soap product containers and associated Gram-negative bacterial growth at CHU-YO, Ouagadougou, Burkina Faso and CNHU-HKM, Cotonou, Benin. Data represent the numbers of samples; within brackets are the number of samples with growth of Gram-negative bacteria. Abbreviations: ABHR = alcohol-based hand rub, PET = polyethylene terephthalate.

Container Type	PET Bottle	Table-Top with Pump Dispenser	Container with Screw Cap/ Dropper Nozzle	Other
CHU-YO (n = 37)				
Numbers	2 (1)	26 (19)	2 (0)	7 (4)
Examples	Soft drink and water bottles	Original ABHR Original scrub	Original povidone iodine Original Dakin Cooper	3 x wall-mounted pump dispensers 4 x screw-cap container, all recycled
Clean/Dirty	Clean: 0 (0) Dirty: 2 (1)	Clean: 2 (1) Dirty: 24 (18)	Clean: 1 (0) Dirty: 1 (0)	Clean: 0 (0) Dirty: 7 (4)
Other observations	All of table-top containers with pump dispensers were overused (scratched surfaces); dispensers were missing in four containers and the dispensers of three containers were broken. Both PET bottles were overused, one had a perforated screw cap.			
CNHU-HKM (n = 32)				
Numbers	12 (9)	15 (13)	4 (4)	Total: 1 (1)
Examples	Soft drink and water bottles	Original ABHR Original antiseptic soap	Original povidone iodine	Non-identifiable uncapped container
Clean/Dirty	Clean: 11 (8) Dirty: 1 (1)	Clean: 15 (13) Dirty: 0 (0)	Clean:3 (3) Dirty: 1 (1)	Clean: 0 (0) Dirty: 1 (1)
Other observations	All table-top containers with pump dispensers had an intact dispenser. They were in use very recently before sampling and were recycled containers of ABHR diffused hospital-wide as part of a hand hygiene project. The original labels were still in place. Six PET bottles had their screw cap intact, four had a perforated cap and two were uncapped.			

**Table 4:** Gram-negative bacterial species isolated from in-use liquid soap samples (n = 51) and associated total colony counts in CHU-YO, Ouagadougou, Burkina Faso and CNHU-HKM, Cotonou, Benin. Total number of species outnumber the total number of samples as in 32 samples, more than one species was isolated. Abbreviation: CFU/mL = colony forming units/mL.

Species	No. of Affected Samples	Associated Total Colony Count (CFU/mL)	Hospital Wards
Enterobacterales, *Aeromonas*/*Vibrio* non-cholerae (n = 14)			
*Enterobacter bugandensis*	1	>10,000	CNHU-HKM Neonatology
*Enterobacter cloacae* complex	3	>10,000	CNHU-HKM Neonatology (n = 2)
			CNHU-HKM Maternity (n = 1)
*Klebsiella oxytoca*	1	>10,000	CHU-YO Surgery
*Klebsiella pneumoniae*	5	>10,000	CNHU-HKM Neonatology (n = 4)
			CNHU-HKM Maternity (n = 1)
*Aeromonas caviae*	2	>10,000	CNHU-HKM Maternity
			CHU-YO Pediatric ward
*Vibrio alginolyticus*	2	>10,000	CHU-YO Internal Medicine
			CNHU-HKM Neonatology
Non-fermentative Gram-negative bacteria (n = 74)			
*Pseudomonas aeruginosa*	14	>10,000	CHU-YO Pediatric ward (n = 1)
			CHU-YO Nephrology-Dialysis (n = 3)
			CHU-YO Surgery (n = 1)
			CNHU-HKM Neonatology (n = 9)
*Pseudomonas* spp. ^a^	21	>10,000 (n = 19) 1001–2500 (n = 2)	CHU-YO Nephrology-Dialysis (n = 2)
			CHU-YO Internal medicine (n = 4)
			CHU-YO Pediatric wards (n = 2)
			CHU-YO Maternity (n = 1)
			CNHU-HKM Neonatology (n = 6)
			CNHU-HKM Pediatric ward (n = 2)
			CNHU-HKM Maternity (n = 3)
			CNHU-HKM Internal medicine (n = 1)
*Acinetobacter* spp. ^b^	6	>10,000 (n = 4) 2501–10,000 (n = 2)	CHU-YO Maternity (n = 1)
			CHU-YO Surgery (n = 3)
			CNHU-HKM Neonatology (n = 1)
			CNHU-HKM Maternity (n = 1)
*Alcaligenes faecalis*	1	>10,000	CHU-YO Neonatology
*Halomonas* spp.	1	>10,000	CNHU-HKM Maternity
*Pannonibacter phragmitetus*	1	>10,000	CNHU-HKM Neonatology
*Shewanella* spp. ^c^	2	>10,000	CHU-YO Pediatric ward
Other Non-fermentative Gram-negative bacteria	28	>10,000 (n = 20) 2501–10,000 (n = 1) 501–1000 (n = 4) <250 (n = 3)	CHU-YO Pediatric ward (n = 5)
			CHU-YO Internal medicine (n = 4)
			CHU-YO Surgery (n = 2)
			CHU-YO Maternity (n = 3)
			CHU-YO Nephrology-Dialysis (n = 1)
			CNHU-HKM Neonatology (n = 3)
			CNHU-HKM Pediatric ward (n = 6)
			CNHU-HKM Maternity (n = 4)

^a^ *Pseudomonas* spp. include *Pseudomonas stutzeri* group (n = 7), *Pseudomonas mendocina* (n = 6), *Pseudomonas putida* group and *Pseudomonas oleovorans* (n = 2 isolates each), *Pseudomonas otitidis* (n = 1), and *Pseudomonas* spp. (n = 3). ^b^ *Acinetobacter* spp. include *Acinetobacter schindleri*, *Acinetobacter junii*, *Acinetobacter pittii*, *Acinetobacter ursingii*, *Acinetobacter haemolyticus* and *Acinetobacter venetianus* (1 isolate each). ^c^ *Shewanella* spp. include *Shewanella decolorationis* and *Shewanella putrefaciens*.

**Table 5:** Gram-negative bacterial species isolated from in-use antiseptics, disinfectants and hand hygiene products other than liquid soap products and associated total colony count in CHU-YO, Ouagadougou, Burkina Faso and CNHU-HKM, Cotonou, Benin. Abbreviations: ABHR = alcohol-based hand rub, CFU/mL = colony forming units/mL, NFGNB = non-fermentative Gram-negative bacteria, QUAT = quaternary ammonium compound.

Affected Product	Gram-Negative Bacterial Species	Total Colony Count (CFU/mL)	Hospital Wards	Comment
Liquid products (n = 7)				
Non-fermentative Gram-negative bacteria (n = 11)				
Chlorhexidine (n = 1)	*Achromobacter xylosoxidans*	2501–10,000	CNHU-HKM Maternity	Recycled container with screw-cap/dropper nozzle
Ethanol (n = 2)	*Pseudomonas putida* group	250–500	CHU-YO Surgery	
	*Pseudomonas putida* group	<250	CHU-YO Internal medicine	Recycled ABHR table-top dispenser
Chlorine (n = 2)	*Acinetobacter baumannii*	>10,000	CHU-YO Pediatric ward	Washing basin with a lid
	*Delftia acidovorans*			Washing basin, no lid
	*Pseudomonas stutzeri*			
QUAT (n = 1)	*Acinetobacter indicus*		CHU-YO Dialysis	Original container
	*Acinetobacter* spp.			
ABHR (n = 1)	*Pseudomonas aeruginosa*		CHU-YO Surgery	Recycled container, no label, no concentration mentioned
	NFGNB			
Bar soap products (n = 4)				
	Enterobacterales (n = 4)			
	*Enterobacter cloacae* complex (n = 2)	>10,000	CHU-YO Neonatology	1 on the bench 1 in a perforated container
	*Enterobacter bugandensis* (n = 1)		CHU-YO Neonatology	No receptacle, put directly on the bench
	*Klebsiella oxytoca* (n = 1)		CHU-YO Surgery	No receptacle, put directly on the bench
Non-fermentative Gram-negative bacteria (n = 3)				
	*Acinetobacter haemolyticus*	>10,000	CHU-YO Surgery	No receptacle, put directly on the bench
	*Wautersiella falsenii*		CHU-YO Neonatology	No receptacle, put directly on the bench
	*Ochrobactrum intermedium*	<250	CHU-YO Surgery	No receptacle, put directly on the bench

## Data Availability

Data supporting the present work results can be found in Supplementary Document S2 submitted with this work.

## Supplementary Materials

The following supporting information can be downloaded at: https://www.mdpi.com/article/10.3390/pathogens12070917/s1, Document S1: Guiding checklist for interview/observation of healthcare workers about antiseptics, disinfectants and soaps; Table S1: Distribution and stock containers (branded bulk products) sampled at CHU-YO, Ouagadougou, Burkina Faso and CNHU-HKM, Cotonou, Benin; Table S2: Antimicrobial resistance profile of Enterobacterales, Aeromonas, and Vibrio non-cholerae isolated from antiseptics, disinfectants and hand hygiene products at CHU-YO, Ouagadougou, Burkina Faso and CNHU-HKM, Cotonou, Benin; Table S3: Antimicrobial resistance profile of non-fermentative Gram-negative bacteria isolated from antiseptics, disinfectants, and hand hygiene products at CHU-YO, Ouagadougou, Burkina Faso and CNHU-HKM, Cotonou, Benin; Table S4: Distribution of clinical isolates in the visited hospital wards at CNHU-HKM, Cotonou, Benin; Table S5: Distribution of clinical isolates in the visited hospital wards at CHU-YO, Ouagadougou, Burkina Faso; Table S6: Quality assessment score of the present cross-sectional survey of Gram-negative growth in antiseptics, disinfectants and hand hygiene products at CHU-YO, Ouagadougou, Burkina Faso and CNHU-HKM, Cotonou, Benin according to the MICRO and ORION-based checklist used in the reference [9].

## Abbreviations

ABHR	Alcohol-based hand rub
AS	Antiseptics
CFU/ml	Colony forming units per ml
CHU-YO	Centre Hospitalier Universitaire Yalgado Ouédraogo
CLSI	Clinical and Laboratory Standards Institute
CNHU-HKM	Centre National Hospitalier Universitaire Hubert Koutoukou Maga
DI	Disinfectants
ESBL	Extended spectrum beta-lactamase
HAI	Healthcare associated infection
HH	Hand hygiene
IPC	Infection prevention and control
LMIC	Low- and middle-income countries
MALDI-TOF	Matrix Assisted Laser Desorption Ionization—Time of Flight
MDR	Multi-drug resistance
NDM	New Delhi metallo-beta-lactamase
NFGNB	Non-fermentative Gram-negative bacteria
PET	Polyethylene terephthalate
TSA	Tryptic soy agar
VIM	Verona Integron-encoded metallo-beta-lactamase

## References

[1] Jacobs J., Hardy L., Semret M., Lunguya O., Phe T., Affolabi D., Yansouni C., Vandenberg O. (2019). Diagnostic Bacteriology in District Hospitals in Sub-Saharan Africa: At the Forefront of the Containment of Antimicrobial Resistance. Front. Med..

[2] World Health Organization (WHO) and the United Nations Children’s Fund (UNICEF) Progress on Wash in Health Care Facilities 2000–2021—Special Focus on WASH and Infection Prevention and Control (IPC). https://www.who.int/publications-detail-redirect/9789240058699.

[3] World Health Organization (WHO) WHO Guideline on Hand Hygiene in Health Care—First Global Patient Safety Challenge Clean Care Is Safer Care. http://whqlibdoc.who.int/publications/2009/9789241597906_eng.pdf.

[4] Dias V.M.C.H., da Silva D.M.W., Burger M., de Iliveira A.A.S., de Capelo P.J., Al E. (2021). Active Surveillance of Carbapenemresistant Gram-Negative Healthcare-Associated Infections in a Low-Middle-Income Country City. Braz. J. Infect. Dis..

[5] Rothe C., Schlaich C., Thompson S. (2013). Healthcare-Associated Infections in Sub-Saharan Africa. J. Hosp. Infect..

[6] World Health Organization (WHO) Member States Information Session on Infection Prevention and Control (IPC). https://apps.who.int/iris/handle/10665/80135.

[7] Kanamori H., Rutala W.A., Weber D.J. (2017). The Role of Patient Care Items as a Fomite in Healthcare-Associated Outbreaks and Infection Prevention. Clin. Infect. Dis..

[8] Lompo P., Heroes A.-S., Agbobli E., Kühne V., Tinto H., Affolabi D., Jacobs J. (2023). Bacterial Contamination of Antiseptics, Disinfectants and Products Used for Hand Hygiene in Healthcare Facilities in High-Income Countries: A Scoping Review. Hygienes.

[9] Lompo P., Agbobli E., Heroes A.-S., vanden Poel B., Kühne V., Kpossou G., Zida A., Halidou T., Dissou A., Jacobs J. (2023). Bacterial Contamination of Antiseptics, Disinfectants and Hand Hygiene Products Used in Healthcare Settings in Low- and Middle Income Countries—A Systematic Review. Hygienes.

[10] Chapman P., Forde B.M., Roberts L.W., Bergh H., Vesey D., Jennison A.V., Moss S., Paterson D.L., Beatson S.A., Harris P.N.A. (2020). Genomic Investigation Reveals Contaminated Detergent as the Source of an Extended-Spectrum-β-Lactamase-Producing *Klebsiella michiganensis* Outbreak in a Neonatal Unit. J. Clin. Microbiol..

[11] Dancer S.J. (1999). Mopping up Hospital Infection. J. Hosp. Infect..

[12] Rutala W.A., Weber D.J. (2004). Disinfection and Sterilization in Health Care Facilities: An Overview and Current Issues. Clin. Infect. Dis..

[13] Weber D.J., Rutala W.A., Sickbert-Bennett E.E. (2007). Outbreaks Associated with Contaminated Antiseptics and Disinfectants. Antimicrob. Agents Chemother..

[14] Weber D.J., Sickbert-Bennett E.E., Kanamori H., Rutala W.A. (2019). New and Emerging Infectious Diseases (Ebola, Middle Eastern Respiratory Syndrome Coronavirus, Carbapenem-Resistant Enterobacteriaceae, *Candida auris*): Focus on Environmental Survival and Germicide Susceptibility. Am. J. Infect. Control.

[15] World Health Organization (WHO) Minimum Requirements for Infection Prevention and Control Programmes. https://www.who.int/publications-detail-redirect/9789241516945.

[16] World Health Organization (WHO) Guidelines on Core Components of Infection Prevention and Control Programmes at the National and Acute Health Care Facility Level. https://www.who.int/teams/integrated-health-services/infection-prevention-control/core-components.

[17] World Bank World Bank Country Classifications by Income Level. https://datahelpdesk.worldbank.org/knowledgebase/articles/906519-world-bank-country-and-lending-groups.

[18] Craven D.E., Moody B., Connoly M.G., Kollisch N.R., Stottmeier K.D., McCabe W.R. (1981). Pseudobacteriemia Caused by Providone-Iodine Solution Contaminated by *Pseudomonas cepacia*. N. Engl. J. Med..

[19] Kampf G., Degenhardt S., Lackner S., Jesse K., von Baum H., Kampf G., McDonald C.O.C. (2014). Poorly Processed Reusable Surface Disinfection Tissue Dispensers May Be a Source of Infection. BMC Infect. Dis..

[20] Assadian O., Kramer A., Christiansen B., Exner M., Martiny H., Sorger A., Suchomel M. (2012). Recommendations and Requirements for Soap and Hand Rub Dispensers in Healthcare Facilities. GMS Krankenhhyg. Interdiszip..

[21] Tomasienwicz D.M., Hotchkiss D.K., Reinbold G.W., Read R.B., Hartman P.A. (1980). The Most Suitable Number of Colonies on Plates for Counting. J. Food Prot..

[22] U.S. Food & Drug Administration, (FDA) BAM Chapter 3: Aerobic Plate Count. https://www.fda.gov/food/laboratory-methods-food/bam-chapter-3-aerobic-plate-count.

[23] Denis F., Ploy M.-C., Martin C., Cattoir V. (2016). Acinetobacter. Bactériologie Médicale—Techniques Usuelles.

[24] Clinical and Laboratory Standards Institute (CLSI) (2023). M100.

[25] Clinical and Laboratory Standards Institute (CLSI) (2016). Methods for Antimicrobial Dilution and Disk Susceptibility Testing of Infrequently Isolated or Fastidious Bacteria.

[26] Clinical and Laboratory Standards Institute (CLSI) (2022). Analysis and Presentation of Cumulative Antimicrobial Susceptibility Test Data.

[27] European Committee on Antimicrobial Susceptibility Testing, (EUCAST) (2023). Expected Resistant Phenotypes.

[28] Magiorakos A.P., Srinivasan A., Carey R.B., Carmeli Y., Falagas M.E., Giske C.G., Harbarth S., Hindler J.F., Kahlmeter G., Olsson-Liljequist B. (2012). Multidrug-Resistant, Extensively Drug-Resistant and Pandrug-Resistant Bacteria: An International Expert Proposal for Interim Standard Definitions for Acquired Resistance. Clin. Microbiol. Infect..

[29] Stone S.P., Cooper B.S., Kibbler C.C., Cookson B.D., Roberts J.A., Medley G.F., Duckworth G., Lai R., Ebrahim S., Brown E.M. (2007). The ORION Statement: Guidelines for Transparent Reporting of Outbreak Reports and Intervention Studies of Nosocomial Infection. Lancet Infect. Dis..

[30] Turner P., Fox-Lewis A., Shrestha P., Dance D.A.B., Wangrangsimakul T., Cusack T.P., Ling C.L., Hopkins J., Roberts T., Limmathurotsakul D. (2019). Microbiology Investigation Criteria for Reporting Objectively (MICRO): A Framework for the Reporting and Interpretation of Clinical Microbiology Data. BMC Med..

[31] Archibald L.K., Shah B., Schulte M., Arduino M.J., Aguero S., Fisher D.J., Stechenberg B.W., Banerjee S.N., Jarvis W.R. (1997). *Serratia marcescens* Outbreak Associated with Extrinsic Contamination of 1% Chlorxylenol Soap. Infect. Control Hosp. Epidemiol..

[32] Buffet-Bataillon S., Rabier V., Bétrémieux P., Beuchée A., Bauer M., Pladys P., Le Gall E., Cormier M., Jolivet-Gougeon A. (2009). Outbreak of *Serratia marcescens* in a Neonatal Intensive Care Unit: Contaminated Unmedicated Liquid Soap and Risk Factors. J. Hosp. Infect..

[33] Villari P., Crispino M., Salvadori A., Scarcella A. (2001). Molecular Epidemiology of an Outbreak of *Serratia marcescens* in a Neonatal Intensive Care Unit. Infect. Control Hosp. Epidemiol..

[34] Fanci R., Bartolozzi B., Sergi S., Casalone E., Pecile P., Cecconi D., Mannino R., Donnarumma F., Leon A.G., Guidi S. (2009). Molecular Epidemiological Investigation of an Outbreak of *Pseudomonas aeruginosa* Infection in an SCT Unit. Bone Marrow Transplant..

[35] Grohskopf L., Roth V., Feikin D., Arduino M., Carson L., JI T., Holt S., Jensen B., Hoffman R., Jarvis W. (2001). *Serratia liquefaciens* Bloodstream Infections from Contamination of Epoetin Alfa at a Hemodialysis Center. N. Engl. J. Med..

[36] Lanini S., D’Arezzo S., Puro V., Martini L., Imperi F., Piselli P., Montanaro M., Paoletti S., Visca P., Ippolito G. (2011). Molecular Epidemiology of a *Pseudomonas aeruginosa* Hospital Outbreak Driven by a Contaminated Disinfectant-Soap Dispenser. PLoS ONE.

[37] Oie S., Arakawa J., Furukawa H., Matsumoto S., Matsuda N., Wakamatsu H. (2012). Microbial Contamination of a Disinfectant-Soaked Unwoven Cleaning Cloth. J. Hosp. Infect..

[38] Rabier V., Bataillon S., Jolivet-Gougeon A., Chapplain J.M., Beuchée A., Bétrémieux P. (2008). Hand Washing Soap as a Source of Neonatal *Serratia marcescens* Outbreak. Acta Paediatr. Int. J. Paediatr..

[39] Sartor C., Jacomo V., Duvivier C., Tissot-Dupont H., Sambbuc R., Drancourt M. (2000). Nosocomial *Serratia marcescens* Infections Associated with Extrinsic Contamination of a Liquid Nonmedicated Soap. Infect. Control Hosp. Epidemiol..

[40] Süer K., Meryem G., Otlu B., Tunç E. (2016). Outbreak of Burkholderia Cepacia Complex Associated with Contaminated Liquid Soap for Hospital Use: A Case Study. Afr. J. Microbiol. Res..

[41] Takahashi H., Kramer M.H., Yasui Y., Fujii H., Nakase K., Ikeda K., Imai T., Okazawa A., Tanaka T., Ohyanna T. (2004). Nosocomial *Serratia marcescens* Outbreak in Osaka, Japan, From 1999 to 2000. Infect. Control Hosp. Epidemiol..

[42] Ben Saida N., Marzouk M., Ferjeni A., Boukadida J. (2009). A Three-Year Surveillance of Nosocomial Infections by Methicillin-Resistant *Staphylococcus haemolyticus* in Newborns Reveals the Disinfectant as a Possible Reservoir. Pathol. Biol..

[43] Khanna A., Khanna M., Aggarwal A. (2013). *Serratia marcescens*—A Rare Opportunistic Nosocomial Pathogen and Measures to Limit Its Spread in Hospitalized Patients. J. Clin. Diagn. Res..

[44] Parasakthi N., Vadivelu J., Ariffin H., Iyer L., Palasubramaniam S., Arasu A. (2000). Epidemiology and Molecular Characterization of Nosocomially Transmitted Multidrug-Resistant *Klebsiella pneumoniae*. Int. J. Infect. Dis..

[45] Stoesser N., Sheppard A.E., Shakya M., Sthapit B., Thorson S., Giess A., Kelly D., Pollard A.J., Peto T.E.A., Walker A.S. (2015). Dynamics of MDR Enterobacter Cloacae Outbreaks in a Neonatal Unit in Nepal: Insights Using Wider Sampling Frames and next-Generation Sequencing. J. Antimicrob. Chemother..

[46] Aktas E., Taspinar E., Alay D., Ögedey E.D.K., Külah C., Comert F. (2010). Extrinsic Contamination of Liquid Soap with Various Gram-Negative Bacteria in a Hospital in Turkey. Infect. Control Hosp. Epidemiol..

[47] Altaher A.M., Ghafoor E.S.A., Amudi W.I., Alderby D.K. (2016). Comparative Identification of Bacterial Quality in Liquid Soap between Nasser and European Gaza Hospitals, Khanyounis Governorate. Asian J. Pharm. Nurs. Med. Sci..

[48] Biswal M., Prasad A., Dhaliwal N., Gupta A.K., Taneja N. (2015). Increase in Hospital Purchase of Hand Hygiene Products: The Importance of Focusing on the Right Product. Am. J. Infect. Control.

[49] Salama A.S.A. (2016). Microbiological Quality of Soaps and Efficacy of Antiseptics and Disinfectants Used in Hospitals in Gaza—Palestine. Master’s Thesis.

[50] Subbannayya K., Bhat G.K., Junu V.G., Shetty S., Jisho M.G. (2006). Can Soaps Act as Fomites in Hospitals?. J. Hosp. Infect..

[51] Zeiny S.M.H. (2009). Isolation of Some Microorganisms from Bar Soaps and Liquid Soaps in Hospital Environments. Iraqi. J. Pharm. Sci..

[52] Eiref S.D., Leitman I.M., Riley W. (2012). Hand Sanitizer Dispensers and Associated Hospital-Acquired Infections: Friend or Fomite?. Surg. Infect..

[53] Espinosa de los Monteros L.E., Silva-Sanchez J., Jiménez L.V., Rojas T., Garza-Ramos U., Valverde V. (2008). Outbreak of Infection by Extended-Spectrum Beta-Lactamase SHV-5-Producing *Serratia marcescens* in a Mexican Hospital. J. Chemother..

[54] Barry M.A., Craven D.E., Goularte T.A., Lichtenberg D.A. (1984). *Serratia marcescens* Contamination of Antiseptic Soap Containing Triclosan: Implications for Nosocomial Infection. Infect. Control.

[55] Jarvis J.D., Wynne C.D., Enwright L., Williams J.D. (1979). Handwashing and Antiseptic-Containing Hospital Soaps In. J. Clin. Pathol..

[56] McBride M.E. (1984). Microbial Flora of In-Use Soap Products. Appl. Environ. Microbiol..

[57] Dolan S.A., Littlehorn C., Glodé M.P., Dowell E., Xavier K., Nyquist A.-C., Todd J.K. (2012). Association of *Bacillus cereus* Infection with Contaminated Alcohol Prep Pads. Infect. Control Hosp. Epidemiol..

[58] Steinhauer K., Meyer B., Ostermeyer C., Rödger H.-J., Hintzpeter M. (2013). Hygienic Safety of Alcohol-Based Hand Disinfectants and Skin Antiseptics. GMS Hyg. Infect. Control.

[59] Cisse M.F., Samb A., Mboup S., Gaye A., David M.P., Sow H.D., Sanokho A. (1987). On a Nosocomial Transmission of *Enterobacter cloacae* in a Tropical Area Children Hospital. Méd. Mal. Infect..

[60] Pinna A., Usai D., Sechi L.A., Zanetti S., Jesudasan N.C.A., Thomas P.A., Kaliamurthy J. (2009). An Outbreak of Post-Cataract Surgery Endophthalmitis Caused by *Pseudomonas aeruginosa*. Ophthalmology.

[61] World Health Organization, (WHO) (2017). Prioritization of Pathogens to Guide Discovery, Research and Development of New Antibiotics for Drug-Resistant Bacterial Infections, Including Tuberculosis.

[62] Ombelet S., Kpossou G., Kotchare C., Agbobli E., Sogbo F., Massou F., Lagrou K., Barbé B., Affolabi D., Jacobs J. (2022). Blood Culture Surveillance in a Secondary Care Hospital in Benin: Epidemiology of Bloodstream Infection Pathogens and Antimicrobial Resistance. BMC Infect. Dis..

[63] Kadri S.S., Adjemian J., Lai Y.L., Spaulding A.B., Ricotta E., Rebecca Prevots D., Palmore T.N., Rhee C., Klompas M., Dekker J.P. (2018). Difficult-to-Treat Resistance in Gram-Negative Bacteremia at 173 US Hospitals: Retrospective Cohort Analysis of Prevalence, Predictors, and Outcome of Resistance to All First-Line Agents. Clin. Infect. Dis..

[64] Yusuf E., Bax H.I., Verkaik N.J., van Westreenen M. (2021). An Update on Eight “New” Antibiotics against Multidrug-Resistant Gram-Negative Bacteria. J. Clin. Med..

[65] Liu S., Xu H., Guo X., Li S., Wang Q., Li Y., Liu R., Gou J. (2021). Emergence and Genetic Characterization of Plasmid-Encoded VIM-2-Producing *Pseudomonas stutzeri* with Novel Integron In1998 Isolated from Cerebrospinal Fluid. Infect. Drug Resist..

[66] Bauer-Savage J., Pittet D., Kim E., Allegranzi B. (2013). Local Production of WHO-Recommended Alcohol-Based Handrubs: Feasibility, Advantages, Barriers and Costs. Bull. World Health Organ..

[67] Kohan C., Ligi C., Dumigan D.G., Boyce J.M. (2002). The Importance of Evaluating Product Dispensers When Selecting Alcohol-Based Handrubs. Am. J. Infect. Control.

[68] Chattman M. (2011). Occurrence of Heterotrophic and Coliform Bacteria in Liquid Hand Soaps from Bulk Refillable Dispensers in Public Facilities. J. Environ. Health.

[69] Momeni S.S., Tomlin N., Ruby J.D. (2015). Isolation of *Raoultella planticola* from Refillable Antimicrobial Liquid Soap Dispensers in a Dental Setting. J. Am. Dent. Assoc..

[70] Gräf W., Kersch D., Scherzer G. (1988). Microbial Contamination of Wall-Attached, One-Way Dispensers of Fluid Soaps. Zbl. Bakt. Hyg..

[71] Otter J.A., Vickery K., Walker J.T., deLancey Pulcini E., Stoodley P., Goldenberg S.D., Salkeld J.A.G., Chewins J., Yezli S., Edgeworth J.D. (2015). Surface-Attached Cells, Biofilms and Biocide Susceptibility: Implications for Hospital Cleaning Anddisinfection. J. Hosp. Infect..

[72] Afolabi B.A., Oduyebo O.O., Ogunsola F.T. (2007). Bacterial Flora of Commonly Used Soaps in Three Hospitals in Nigeria. East Afr. Med. J..

[73] Danchaivijitr S., Dhiraputra C., Rongrungruang Y., Srihapol N., Pumsuwan V. (2005). Microbial contamination of antiseptics and disinfectants. Microb. Contam. Antiseptics Disinfect..

[74] Gajadhar T., Lara A., Sealy P., Adesiyun A.A. (2003). Microbial Contamination of Disinfectants and Antiseptics in Four Major Hospitals in Trinidad. Rev. Panam. Salud Publica/Pan Am. J. Public Health.

[75] Allegranzi B., Nejad S.B., Combescure C., Graafmans W., Attar H., Donaldson L., Pittet D. (2011). Burden of Endemic Health-Care-Associated Infection in Developing Countries: Systematic Review and Meta-Analysis. Lancet.

[76] McNaughton M., Mazinke N., Thomas E. (1995). Newborn Conjuctivitis Associated with Triclosan 0.5% Antiseptic Intrinsically Contaminated with *Serratia marcescens*. Can. J. Infect. Control.

[77] Zapka C.A., Campbell E.J., Maxwell S.L., Gerba C.P., Dolan M.J., Arbogast J.W., Macinga D.R. (2011). Bacterial Hand Contamination and Transfer after Use of Contaminated Bulk-Soap-Refillable Dispensers. Appl. Environ. Microbiol..

[78] Blanc D.S., Gomes Magalhaes B., Abdelbary M., Prod’hom G., Greub G., Wasserfallen J.B., Genoud P., Zanetti G., Senn L. (2016). Hand Soap Contamination by *Pseudomonas aeruginosa* in a Tertiary Care Hospital: No Evidence of Impact on Patients. J. Hosp. Infect..

[79] Mapping Antimicrobial Resistance and Antimicrobial Use Partnership Measure Levels of Antimicrobial Resistance (AMR) and Antimicrobial Use (AMU) Based on Existing, Historical, Records, Assess the Relationship between the Two and Provide Policy Recommendations to Strengthen AMR and Antimicrobial Consumption (AMC) Surveilla. https://aslm.org/what-we-do/maap/.

[80] Centers for Disease Control and Prevention, (CDC) (2002). Guideline for Hand Hygiene in Health-Care Settings Recommendations of the Healthcare Infection Control Practices Advisory Committee and the HICPAC/SHEA/APIC/IDSA Hand Hygiene Task Force. Morb. Mortal. Wkly. Rep. Recomm..

[81] Schaffner D.W., Jensen D., Gerba C.P., Shumaker D., Arbogast J.W. (2018). Influence of Soap Characteristics and Food Service Facility Type on the Degree of Bacterial Contamination of Open, Refillable Bulk Soaps. J. Food Prot..

[82] Caetano J.A., Lima M.A., Miranda M.D.C., Serufo J.C., Ponte P.R.L. (2011). Identification of Bacterial Contamination in Liquid Soap for Hospital Use. Rev. Esc. Enferm..

