# Supplementary material for: Growth of Gram-Negative Bacteria in Antiseptics, Disinfectants and Hand Hygiene Products in Two Tertiary Care Hospitals in West Africa—A Cross-Sectional Survey
Source: Pathogens. 2023 Jul 7;12(7):917. doi: 10.3390/pathogens12070917 (PMC10384974; doi:10.3390/pathogens12070917)
Supplement: Supplementary file 1 [file pathogens-12-00917-s001.zip › PL Document S1.pdf]

191215 – Antiseptics-disinfectants-soaps questions from production to use_AH_LP

Hospital: ………
Product: ……e.g. *Dakin, povidone iodine 10%, ethanol, soap, eosin, soap*
Brand: …….

Production

Produced in-house or bought from manufacturer?

If produced in-house,

- Who is responsible for preparation?
- What:
  - Which stock products used (brand, concentration; if water, which water source)?
  - Which volumes are made?
  - Which containers and tools are used for stock preparation?
  - What is the final stock solution concentration?
- Where is stock solution prepared?
- When: what frequency?
- How: What is the preparation procedure? Written procedure available?

Labelling: if yes:

- What information is written on the label?
- What kind of label? (written on container, paper label, bandage, …)

Reuse of containers? If yes, which cleaning procedures for containers?

- Who?
- Where?
- When?
- How?

Storage

- What:
  - Which volume?
  - Which containers? (transparent, opaque, opened, closed)
- Where are products stored?
- When: Storage time?
- How:
  - Dedicated space
  - Temperature monitored?
  - Temperature
  - Humidity
  - Light/sunray
  - Cleanness
  - Windows
  - Ventilation

Dilution

Are stock solutions diluted before distribution? If yes:

- Who is responsible for preparation?
- What:
  - Which dilution factor – what is final concentration?
  - Which stock products used (brand, concentration)?
  - Which final volumes are made?
  - Which containers and tools are used for stock preparation?
- Where is stock solution prepared?
- When: frequency?
- How: What is the preparation procedure? (Written procedure available?)

Labelling: if yes:

- What information is written on the label?
- What kind of label? (written on container, paper label, bandage, …)

Reuse of containers? If yes, which cleaning procedures for containers?

- Who?
- Where?
- When?
- How?

Distribution

- Who is responsible for distribution?
- What:
  - Which container is distributed to the wards + which volume?
  - Which procedure of aliquoting is used, if applicable?
- Where are products distributed to?
- When: What is the frequency of distribution to the wards?
- How?

Use (table per ward)

- Is liquid diluted or adapted in the ward, after distribution? If yes, see questions above.
- Who
  - Who manages the antiseptics/disinfectants/soaps at the ward?
  - Who uses the antiseptics/disinfectants/soaps at the ward?
- What:
  - How many bottles are available at the ward?
  - What is the product used for (antisepsis, disinfection, other; + details)?
- When: What is turnover time at the wards?
- How:
  - What other aids are used during product use? (bandages, tweezer, cotton, gauze, cloth, …)
  - How are the antiseptics/disinfectants/soaps applied to these aids?
-

What is done with the container after use? (trash, reuse)

- If reused, what is the cleaning program?
  - Who?

- Where?
- When?
- How?

Draw schematic flow of product in the hospital
