# Supplementary material for: Growth of Gram-Negative Bacteria in Antiseptics, Disinfectants and Hand Hygiene Products in Two Tertiary Care Hospitals in West Africa—A Cross-Sectional Survey
Source: Pathogens. 2023 Jul 7;12(7):917. doi: 10.3390/pathogens12070917 (PMC10384974; doi:10.3390/pathogens12070917)
Supplement: Supplementary file 1 [file pathogens-12-00917-s001.zip › Table S1.pdf]

Table S1. Distribution and stock containers (branded bulk products) sampled at CHU-YO, Ouagadougou, Burkina Faso and CNHU-HKM, Cotonou, Benin. Data represent number of products sampled; between brackets are the numbers of products with growth of Gram-negative bacteria. See Box 1 for definitions. Number of isolates (n = 15) outnumber number of samples with growth of Gram-negative bacteria (n = 13) as two samples yielded two different species each. Abbreviations: ABHR = alcohol-based hand rub, CFU/ml = colony forming units/ml, NFGNB = non-fermentative Gram-negative bacteria, QUAT = quaternary ammonium compound.

**Products**	**Distribution containers**	**Stock containers**		**Growth of Gram-negative bacteria – association with total colony counts (CFU/ml)**
		**In-use**	**Sealed**	
Liquid soap	8 (4)	9 (6)	2	*Distribution containers:*<br>Four samples grew NFGNB (7 isolates, total colony counts > 10,000 CFU/ml) and *Enterobacter cloacae* complex and *Klebsiella* spp. (1 isolate each, > 10,000 CFU/ml).<br>Samples were obtained in CNHU-HKM neonatology (n = 2, including the sample with *E. cloacae* complex and *Klebsiella* spp.), CNHU – HKM central stock (n = 1) and CHU-YO surgery (n = 1)<br>*Stock containers:*<br>Six samples (all open) grew NFGB (n = 5 not identified by MALDI-TOF, 1 *Acinetobacter schindleri*, all but one with total colony counts > 10,000/ml. Samples were obtained at CHU-YO internal medicine, maternity, surgery and neonatology.
Chlorine	7 (1)	1	-	One intermediate container grew with *P. stutzeri* group, < 250 CFU/ml
Ethanol 70%	21 (2)	-	-	One intermediate container grew with *Acinetobacter schindleri* and *Acinetobacter* spp. (total colony count > 10,000 CFU/ml, CHU-YO dialysis). Another sample grew *Pantoea dispersa* (total colony count < 250 CFU/ml, CNHU-HKM neonatology).
Dakin	4	-	-	
Chlorhexidine	-	-	1	
Iodine tincture	1	-	-	
QUAT	-	1	1	
ABHR	-	-	1	
**Total**	**41 (7)**	**11 (6)**	**5 (0)**	None of the sealed samples grew with Gram-negative bacteria
