# Supplementary material for: Growth of Gram-Negative Bacteria in Antiseptics, Disinfectants and Hand Hygiene Products in Two Tertiary Care Hospitals in West Africa—A Cross-Sectional Survey
Source: Pathogens. 2023 Jul 7;12(7):917. doi: 10.3390/pathogens12070917 (PMC10384974; doi:10.3390/pathogens12070917)
Supplement: Supplementary file 1 [file pathogens-12-00917-s001.zip › Table S2.pdf]

Table S2. Antimicrobial resistance profile of Enterobacterales, *Aeromonas* and *Vibrio* non-cholerae isolated from antiseptics, disinfectants and hand hygiene products at CHU-YO, Ouagadougou, Burkina Faso and CNHU-HKM, Cotonou, Benin. Numbers refer to the number of resistant isolates among the total tested for each antibiotic. One isolate of *Klebsiella oxytoca* was not available for antimicrobial susceptibility testing. Abbreviations: ESBL = extended spectrum beta-lactamase, MDR = multidrug resistant.

**Antibiotics**	**Enterobacterales (n = 16)**	***Aeromonas* spp. and *Vibrio* non-cholerae (n = 4)**
Ampicillin	14	2
Temocillin	1	-
Amoxicillin-clavulanic acid	10	0
Piperacillin-tazobactam	3	0
Cefuroxime	10	0
Ceftriaxone	7	0
Ceftazidime	6	0
Meropenem	0	0
Gentamicin	5	0
Amikacin	0	0
Trimethoprim-sulfamethoxazole	12	1
Ciprofloxacin	9	0
Tetracycline	-	0
ESBL production	8	-
MDR	9	-
