# Supplementary material for: Growth of Gram-Negative Bacteria in Antiseptics, Disinfectants and Hand Hygiene Products in Two Tertiary Care Hospitals in West Africa—A Cross-Sectional Survey
Source: Pathogens. 2023 Jul 7;12(7):917. doi: 10.3390/pathogens12070917 (PMC10384974; doi:10.3390/pathogens12070917)
Supplement: Supplementary file 1 [file pathogens-12-00917-s001.zip › Table S3.pdf]

Table S3. Antimicrobial resistance profile of non-fermentative Gram-negative bacteria isolated from antiseptics, disinfectants and hand hygiene products at CHU-YO, Ouagadougou, Burkina Faso and CNHU-HKM, Cotonou, Benin. Numbers refer to the numbers of resistant isolates among the total tested for each antibiotic. One isolate of *Pseudomonas aeruginosa* and four isolates of *Pseudomonas* spp. were not available for antimicrobial susceptibility testing. Abbreviations: NDM = New Delhi metallo-β-lactamase, VIM = Verona Integron-encoded Metallo-beta-lactamase

**Antibiotics**	***Pseudomonas aeruginosa* (n = 14)**	***Pseudomonas* spp. (n = 25)**	***Acinetobacter* spp. (n = 12)**
Piperacillin-tazobactam	0	3	2
Ceftriaxone	-	-	3
Ceftazidime	0	1	3
Gentamicin	1	1	2
Amikacin	0	0	2
Meropenem	0	1 (VIM)	1 (NDM)
Ciprofloxacin	0	0	2
Trimethoprim-sulfamethoxazole	-	--	4
Tetracycline	-	-	3
Doxycycline	-	-	1
MDR	0	0	3
