# Supplementary material for: Growth of Gram-Negative Bacteria in Antiseptics, Disinfectants and Hand Hygiene Products in Two Tertiary Care Hospitals in West Africa—A Cross-Sectional Survey
Source: Pathogens. 2023 Jul 7;12(7):917. doi: 10.3390/pathogens12070917 (PMC10384974; doi:10.3390/pathogens12070917)
Supplement: Supplementary file 1 [file pathogens-12-00917-s001.zip › Table S4.pdf]

Table S4. Distribution of clinical isolates in the visited hospital wards at CNHU-HKM, Cotonou, Benin. Data represent the numbers of deduplicated isolates from blood cultures collected in the wards from which antiseptics, disinfectants and hand hygiene products were sampled in a period six month before to six months after the cross-sectional survey (July 2019 –June 2020); isolates were identified by conventional methods, MALDI-TOF and a commercial system (API 20E and API 20NE, bioMérieux, Marcy L'Etoile, France).

Species isolated from blood cultures	Internal medicine	Maternity	Neonatology	Pediatrics	Total
**Enterobacterales (n = 97)**					
Klebsiella pneumoniae	-	-	27	25	52
Klebsiella spp.	-	-	2	-	2
Enterobacter cloacae complex	3	-	6	15	24
Escherichia coli	1	1	9	6	17
Salmonella spp.	-	-	-	2	2
**Non-fermentative Gram-negative bacteria (n = 60)**					
Burkholderia cepacia complex	-	2	6	20	28
Acinetobacter spp.	-	-	3	11	14
Acinetobacter baumannii /calcoaceticus complex	-	-	2	6	8
Pseudomonas aeruginosa	-	-	2	3	5
Pseudomonas stutzeri	-	-	-	1	1
Pseudomonas putida	-	-	1	-	1
Stenotrophomonas maltophilia	-	-	-	1	1
Ochrobactrum spp.	-	-	-	1	1
Rhizobium radiobacter	-	-	1	-	1
**Gram-positive cocci (n = 15)**					
Staphylococcus aureus	2	-	2	9	13
Staphylococcus saprophyticus	-	-	1		1
Streptococcus spp.	-	-	-	1	1
**Fungi (n = 2)**					
Candida rugosa	-	-	-	1	1
Kodamaea ohmeri	-	-	-	1	1
**Total**	**6**	**3**	**62**	**103**	**174**
