# Supplementary material for: Growth of Gram-Negative Bacteria in Antiseptics, Disinfectants and Hand Hygiene Products in Two Tertiary Care Hospitals in West Africa—A Cross-Sectional Survey
Source: Pathogens. 2023 Jul 7;12(7):917. doi: 10.3390/pathogens12070917 (PMC10384974; doi:10.3390/pathogens12070917)
Supplement: Supplementary file 1 [file pathogens-12-00917-s001.zip › Table S5.pdf]

Supplementary Table 5. Distribution of clinical isolates in the visited hospital wards at CHU-YO, Ouagadougou, Burkina Faso. Data represent the numbers of deduplicated isolates from blood cultures collected in the wards from which antiseptics, disinfectants and hand hygiene products were sampled in a period of six months before to six months after the cross-sectional survey (January 2019 –January 2020); isolates were identified by MALDI-TOF.

Species isolated from blood cultures	Internal medicine	Maternity	Neonatology	Pediatrics	Surgery	Nephrology-Dialysis	Other	Total
**Enterobacterales (n = 59)**								
Klebsiella pneumoniae	2	2	1	17	-	-	4	26
Escherichia coli	5	1	-	10	1	2	2	21
Enterobacter cloacae	-	-	-	2	-	-	2	4
Enterobacter bugandensis	2	-	-	1	-	-	-	3
Morganella morganii	-	-	-	1	-	-	1	2
Pantoea spp.	-	-	-	1	-	-	-	1
Mixta (Pantoea) calida	-	-	-	1	-	-	-	1
Salmonella spp.	-	-	-	1	-	-	-	1
**Non-fermentative Gram-negative bacteria (n = 26)**								
Acinetobacter baumannii	-	-	1	5	-	-	1	7
Acinetobacter nosocomialis	-	-	-	1	-	-	-	1
Acinetobacter spp.	-	-	-	3	-	-	-	3
Pseudomonas aeruginosa	1	-	-	4	-	-	1	6
Pseudomonas stutzeri	-	-	-	1	-	-	-	1
Aeromonas spp.	-	-	-	-	-	1	-	1
Burkholderia cepacia complex	-	-	-	2	-	-	-	2
Burkholderia pseudomallei	-	-	-	1	-	-	-	1
Moraxella spp.	-	-	-	1	-	-	-	1
Other	-	-	-	2	1	-	-	3
**Gram-positive cocci (n = 28)**								
Enterococcus faecalis	-	-	-	3	-	1	-	4
Enterococcus faecium	-	-	-	8	-	1	-	9
Staphylococcus aureus	2	-	1	8	1	3	-	15
**Fungi (n = 4)**								
Candida albicans	-	-	-	1	-	-	-	1
Candida spp.	-	-	-	1	-	-	-	1
Candida tropicalis	-	-	-	1	-	1	-	2
**Total**	**12**	**3**	**3**	**76**	**2**	**9**	**11**	**117**
