# Supplementary material for: Growth of Gram-Negative Bacteria in Antiseptics, Disinfectants and Hand Hygiene Products in Two Tertiary Care Hospitals in West Africa—A Cross-Sectional Survey
Source: Pathogens. 2023 Jul 7;12(7):917. doi: 10.3390/pathogens12070917 (PMC10384974; doi:10.3390/pathogens12070917)
Supplement: Supplementary file 1 [file pathogens-12-00917-s001.zip › Table S6.pdf]

Table S6. Quality assessment score of the present cross-sectional survey of Gram-negative growth in antiseptics, disinfectants and hand hygiene products at CHU-YO, Ouagadougou, Burkina Faso and CNHU-HKM, Cotonou, Benin according to the MICRO and ORION-based checklist used in the reference [9]. The colors refer to the categories of “green = good”, “yellow = satisfactory”, and “red = poor” quality for each selected item. Abbreviations: MICRO = Microbiology Investigation Criteria for Reporting Objectively, ORION = Outbreak Reports and Intervention studies Of Nosocomial infection

	**Item scored**	**Justification of the score**	**Score**
Title/abstract	Title and Abstract provided the information about "outbreak" or "cross-sectional survey"	Title and Abstract mention that the article is about a cross-sectional survey	🟢
Introduction	The background/rationale of the study and the primary and secondary objectives were well structured	The Background section of the manuscript summarizes the previous studies done worldwide and particularly in low- and middle-income countries, and points to the gaps in the existing knowledge. The primary and the secondary objectives are described in detail.	🟢
Study design/setting	Healthcare setting was well described	The hospitals of the survey are well described - hospital level and wards, type of centers (public, private…)	🟢
Samples collected	Product and active ingredient were provided	Ingredients of the liquid soap products were not known at the study sites and not retrievable	🟡
	The use/application of the product(s) was well described	The indications of use of the products were listed during ward visits. For some products, multiple applications were mentioned, also beyond the intended use – this is mentioned in the text.	🟡
	A correct terminology was used (antiseptics, disinfectants, hand hygiene products)	Terminology of products (antiseptics, disinfectant, alcohol-based hand rub and liquid soap) was appropriate (in line with product identity and intended use)	🟢
	In cross-sectional surveys, sample selection and numbers (denominators) were provided	Sample selection is systematically and well described (all antiseptics, disinfectants and soap products observed during ward visits) and representative (denominators (numbers of beds and wards) are provided.	🟢
	In cross-sectional surveys, sufficiently large sample sizes were provided	Sample sizes are sufficiently large for in-use liquid soap and alcohol-based products to support findings and conclusions about comparisons, but fewer for other products and stock and distribution containers (the latter being a secondary objective). Observations and data from interviews were sufficient to trace the risk factors.	🟡
	Microbiological culture methods used were appropriate	We used a consistent sample technique (surface and deeper content), quantitative culture methods, neutralizer-containing plate count agar and selective media for Gram-negative bacteria, microbiology methods are well described.	🟢

	Antibiotic susceptibility testing methods were appropriate	Antibiotic susceptibility testing correctly described (methods with interpretative standard, correct panel of antibiotics used) and reported, and detection of Multidrug Resistance is well described.	
	Additional investigations were done and well reported (interview, questionnaire, review of procedures)	Questionnaire, interview with staff, observations, review of procedures were done and reported. A structured guidance list was used (Supplementary Document 1).	
Results	Reporting of Results was complete and appropriate	Results are consistently reported according to the Methods section and with appropriate details and denominators.	
	Risk factors were assessed	Risk factors along the life cycle of products (defined in a previous review) were retrieved and explored by observations, interviews with staff and culture of stock and intermediate containers.	
Discussion	Key results, limitations and strength, interpretation and comparison with literature, and generalizability	The discussion comprises the summary of main findings, limitations and strengths, comparison with previously published findings, explanations, relevance and generalizability.	
